# Application of a Hybrid Forest Growth Model to Evaluate Climate Change Impacts on Productivity, Nutrient Cycling and Mortality in a Montane Forest Ecosystem

**DOI:** 10.1371/journal.pone.0135034

**Published:** 2015-08-12

**Authors:** Brad Seely, Clive Welham, Kim Scoullar

**Affiliations:** 1 Department of Forest Resources Management, University of British Columbia, Vancouver, British Columbia, Canada; 2 Life Sciences Programming Incorporated, Naramata, British Columbia, Canada; University of California Davis, UNITED STATES

## Abstract

Climate change introduces considerable uncertainty in forest management planning and outcomes, potentially undermining efforts at achieving sustainable practices. Here, we describe the development and application of the FORECAST Climate model. Constructed using a hybrid simulation approach, the model includes an explicit representation of the effect of temperature and moisture availability on tree growth and survival, litter decomposition, and nutrient cycling. The model also includes a representation of the impact of increasing atmospheric CO_2_ on water use efficiency, but no direct CO_2_ fertilization effect. FORECAST Climate was evaluated for its ability to reproduce the effects of historical climate on Douglas-fir and lodgepole pine growth in a montane forest in southern British Columbia, Canada, as measured using tree ring analysis. The model was subsequently used to project the long-term impacts of alternative future climate change scenarios on forest productivity in young and established stands. There was a close association between predicted sapwood production and measured tree ring chronologies, providing confidence that model is able to predict the relative impact of annual climate variability on tree productivity. Simulations of future climate change suggest a modest increase in productivity in young stands of both species related to an increase in growing season length. In contrast, results showed a negative impact on stemwood biomass production (particularly in the case of lodgepole pine) for established stands due to increased moisture stress mortality.

## Introduction

Worldwide, forest health and productivity are being affected by anthropogenic climate change. The frequency and intensity of catastrophic natural disturbance agents, for example, are rising [1–4]. Moreover, evidence suggests that unusually severe drought events have triggered a significant rise in mortality rates in forested regions throughout the world [5, 6]. Climate change is expected to influence long-term forest productivity through its effect on moisture availability [7, 8], temperature-limited net photosynthetic rates [9, 10] and nutrient cycling [11]. After litter quality, temperature and soil moisture are the key determinants of heterotrophic respiration and nutrient mineralization rates in temperate forests [12, 13].

Efforts at modeling the impact of climate on forests can be divided into three broad categories: dynamic global vegetation models (DGVMs), statistical models derived from climate envelope analysis, and stand-level, process-based models. The overall objective of DGVMs is to evaluate the influence of climate on the biogeochemical and hydrological processes regulating vegetation growth dynamics [14]. Although they can be detailed with respect to process simulation, these models are designed to predict general patterns of vegetation development over large spatial and temporal scales [15] and have limited application for evaluating alternative forest adaptation strategies (see, for example, [16]). In general, DGVMs may be best suited to evaluating the impact of changing climate regimes on regional patterns of forest productivity and hydrology.

Models based on the climate envelope approach rely on detailed statistical analyses of historical climate data collected from a species’ observed range. Deviations from climate normals are calculated for future climate scenarios and then used to project changes in growth rates, mortality, and species distributions. Examples include a modified version of the Forest Vegetation Simulator (FVS) model [17], and adjustments to site index based on anticipated changes in growing-degree days [18]. The strength of this approach is that it provides a method for predicting climate change impacts with relatively small calibration data requirements. Challenges with the approach include, 1) limited insight with respect to the underlying mechanisms of response, 2) an assumption that current species distributions are dictated exclusively by climate, and 3) the issue that if the range of variation in future climate exceeds the historical climate regime then applications of the model are beyond the scope of its statistical foundations.

The third category of model is the stand-level, process-based models. These models employ physiological and physical principles in conjunction with simulated edaphic conditions to project forest development and productivity under a changing climate. They vary widely in their complexity and application, from comprehensive, research-oriented ecosystem models (e.g. Ecosys; [19]) to less complex, management-oriented models such as CABALA [20] to relatively simplified models designed for broad application such as 3PG [21]. The more complex models can be difficult to calibrate because they usually comprise many site and species-specific parameters. This can necessitate expensive, multi-year field research programs to support their application (e.g. [20]). Highly simplified process models usually have lower calibration requirements but they often cannot adequately address the complexity of forest management in the face of climate change. A compromise approach is embodied in ‘hybrid’ process-based models, in which empirical data inputted to the model are used to ‘self-calibrate’ at least some of the algorithm parameters associated with ecosystem processes (see [22]). This makes it possible to retain adequate model complexity while minimizing the calibration load [23].

Climate change introduces considerable uncertainty into forest management planning and outcomes, potentially undermining efforts at achieving sustainable practices [24, 25]. There is a need for models capable of projecting the potential impact of climate change on long-term patterns of forest growth and development while being reasonably accessible to forest managers. Ideally, such models should: 1) be capable of representing the effects of climate change on forest productivity and drought-related mortality, 2) include the ability to simulate a variety of forest management options to allow for an evaluation of adaptive management strategies, 3) provide broad outputs relevant to multi-objective forest management, and 4) be relatively straightforward to calibrate.

Here we describe the development and application of the hybrid simulation model FORECAST Climate. The model is evaluated with respect to its ability to reproduce the effects of historic annual climate variability on the annual variation of Douglas-fir and lodgepole pine growth in a montane forest located in southern British Columbia, Canada as measured using tree ring analysis. Simulations of the long-term impacts of alternative future climate change scenarios on forest productivity in young and established stands were conducted with the verified model.

## Methods

### 2.1. Model Description

FORECAST Climate was developed as an extension of FORECAST [26] which is a stand-level, management-oriented forest growth simulator. FORECAST was designed to accommodate a wide variety of harvesting and silvicultural systems in order to compare and contrast their effect upon forest productivity, stand dynamics and a series of biophysical indicators of non-timber stand values. The model has been used in a wide variety of applications and has been evaluated against field data for growth, yield, ecophysiological and soil variables [27–30].

FORECAST is a ‘hybrid’ model and many of its parameters are calibrated using historical bioassay data inputted to the model. As with any model of this type, a fundamental assumption with respect to its successful calibration and application is that the past climate regime constitutes a faithful representation of future climate conditions. The validity of this assumption is now questionable given anticipated trends in greenhouse gas emissions and their associated impacts on future temperature and precipitation patterns [31]. The FORECAST model simulates the effect of light and nutrient availability on forest productivity but it has no explicit representation of moisture or temperature on ecosystem processes. Thus, the rationale in developing FORECAST Climate was to incorporate these two factors. The foundation for FORECAST Climate was established through the creation of a dynamic linkage with a forest hydrology model including direct feedback to the core processes driving forest ecosystem production.

#### 2.1.1. The basic FORECAST model

**Fig 1:**
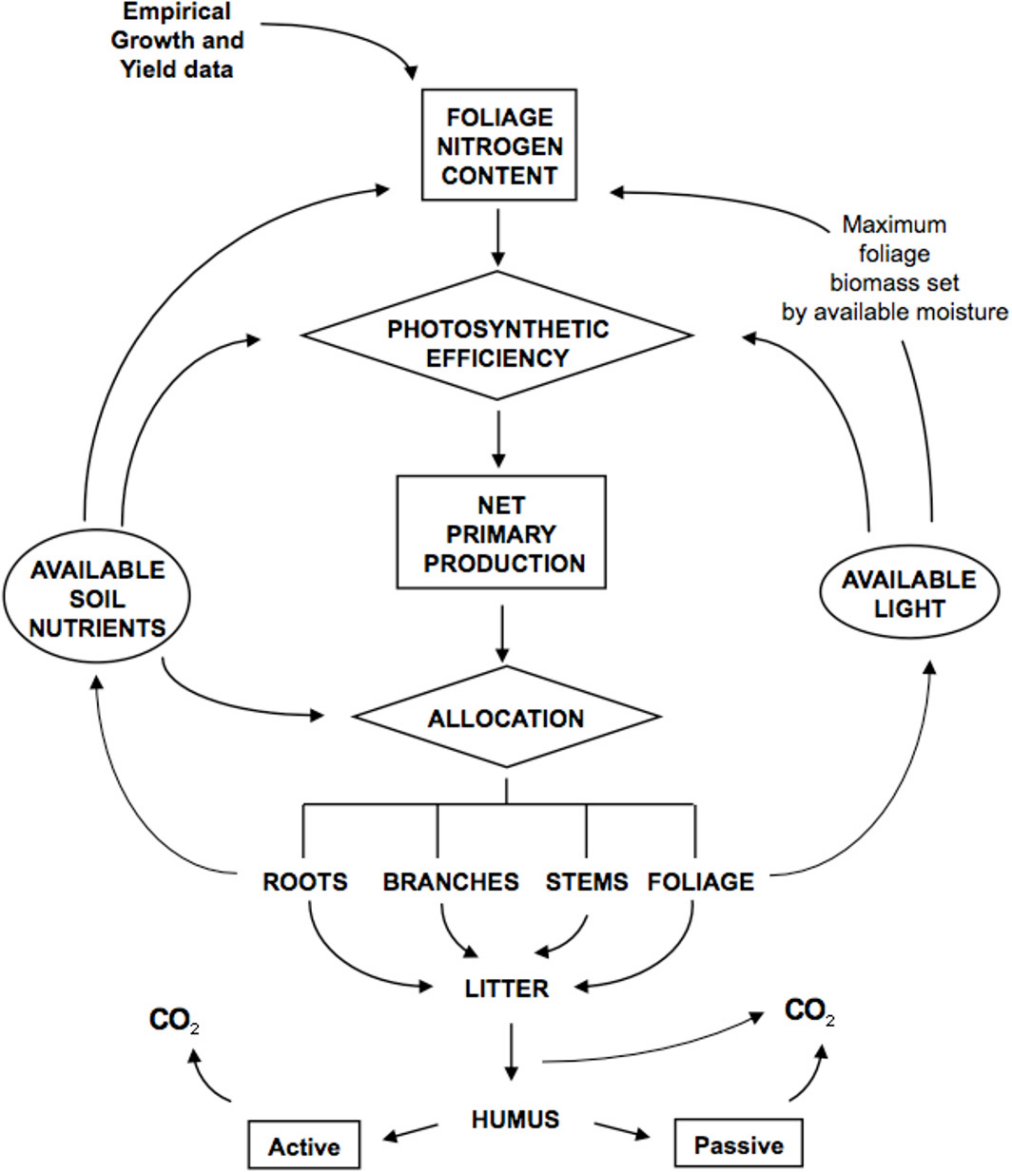
Schematic illustration of the key ecosystem processes and flows represented in FORECAST (after [30]).

In FORECAST, the rates of key ecosystem processes are combined with data describing rates of decomposition, nutrient cycling, light competition, and other ecosystem properties to simulate annual changes in forest growth (Fig 1). Plant populations can be initiated from seed and/or vegetatively, and stand development can occur with or without competition from non-target tree species and understory populations. Decomposition and dead organic matter dynamics are simulated using a method in which specific biomass components (eg. foliage, fine roots, branches, etc.) are transferred at the time of tissue death to one of a series of independent litter types. Residual litter mass and its associated nutrient content are transferred to active and passive humus pools when the mass remaining is within 15–20% of the original litter mass. Typically, mean residence times for active and passive humus types are 50 and 600 years, respectively. See [26] for further details.

#### 2.1.2. The ForWaDy Model

**Fig 2:**
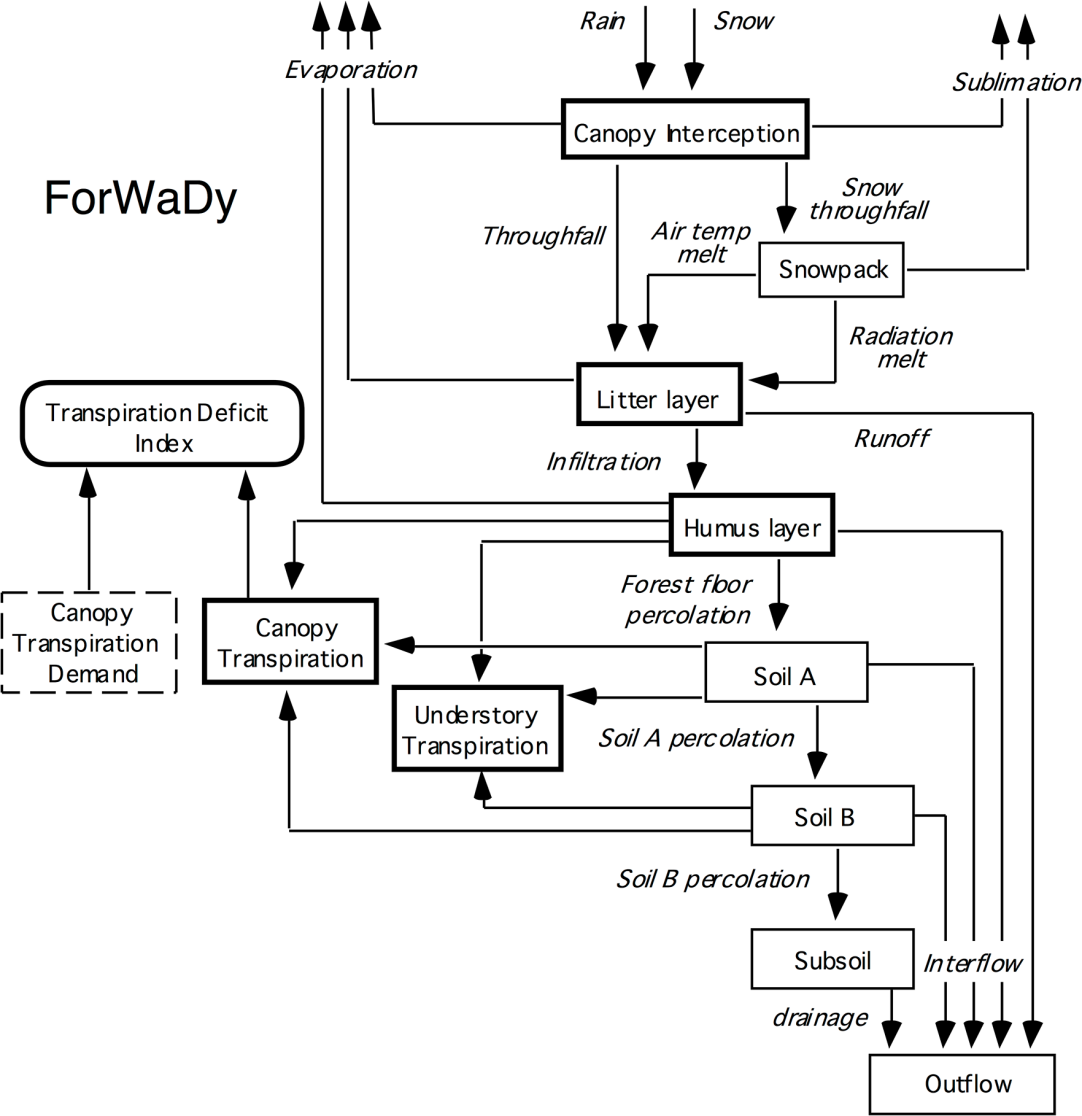
Schematic diagram of the ForWaDy model indicating water flow pathways and storage compartments.

The Forest Water Dynamics (ForWaDy; [32]) model simulates the hydrologic dynamics of a forest stand on a daily time step for a given set of climatic and vegetation conditions. The model was specifically designed with a structure to facilitate its linkage to FORECAST [32]. It has been shown to perform well for predicting the effect of forest management on evapotranspiration [33] and temporal patterns in soil moisture content under field conditions [33, 34]. The model represents potential evapotranspiration (PET) using an energy balance approach based on a modified version of the Priestly-Taylor equation [35]. This equation is effective in predicting evapotranspiration under a wide variety of forest types and conditions [36–39]. Net shortwave solar radiation interception is used to drive the PET calculations. It is calculated for each tree and plant species from a light competition submodel built into FORECAST (see [26]) and surface albedo. ForWaDy includes a representation of the vertical flow of water through canopy and soil layer compartments (Fig 2). Movement of water through each soil layer is regulated by its physical properties that dictate moisture holding capacity, permanent wilting point moisture content, and infiltration rate.

Water stress is calculated for each species separately on a daily time step and is expressed as a transpiration deficit index (TDI). The TDI is the relative difference between potential energy-limited transpiration demand and actual transpiration:

$${\text{TDI}}_{\text{i},\text{d}}=\left({\text{CanT}}_{\text{Demand},\;\text{i},\text{d}}-{\text{CanT}}_{\text{Actual},\;\text{i},\text{d}}\right)/\;{\text{CanT}}_{\text{Demand},\;\text{i},\text{d}}$$ (1)

where:

CanT_Demand, i,d_ = energy-driven transpiration demand for species *i* (mm) on day *d*, as a function of leaf area index, intercepted short-wave radiation, canopy albedo, and canopy resistance,

CanT_Actual, i,d_ = actual tree transpiration for species *i* (mm) on day *d*, as a function of CanT_Demand i,d_, root occupancy, and available soil moisture.

A detailed description of the ForWaDy model is provided in the S1 File. Its general data requirements are listed in Table 1.

**Table 1:** General data requirements for the ForWaDy model.

Climate data (daily)	Vegetation data	Forest floor & soil data
mean, max and min air temperature (°C)	seasonal conifer and hardwood LAI	fine litter mass (kg/ha)
solar radiation^1^ (MJ/m^2^)	seasonal understory % cover	humus layer depth (cm) and bulk density (g/cm^3^)
total precipitation (mm)	rooting depths for trees (cm)	depth of mineral soil layers (cm)
snow fraction	rooting depths for understory (cm)	soil texture^3^ (by layer)
atmospheric [CO_2_]^2^	canopy resistance and albedo (by species)	coarse fragment content (% by layer)

^1^ Solar radiation may be estimated from max and min air temperature, elevation, latitude, slope and aspect using published radiation models.

^2^ Only annual data required.

^3^ Standard texture classes used to estimate clay content.

#### 2.1.3. Simulating the effect of increasing CO2 on forest growth

**Fig 3:**
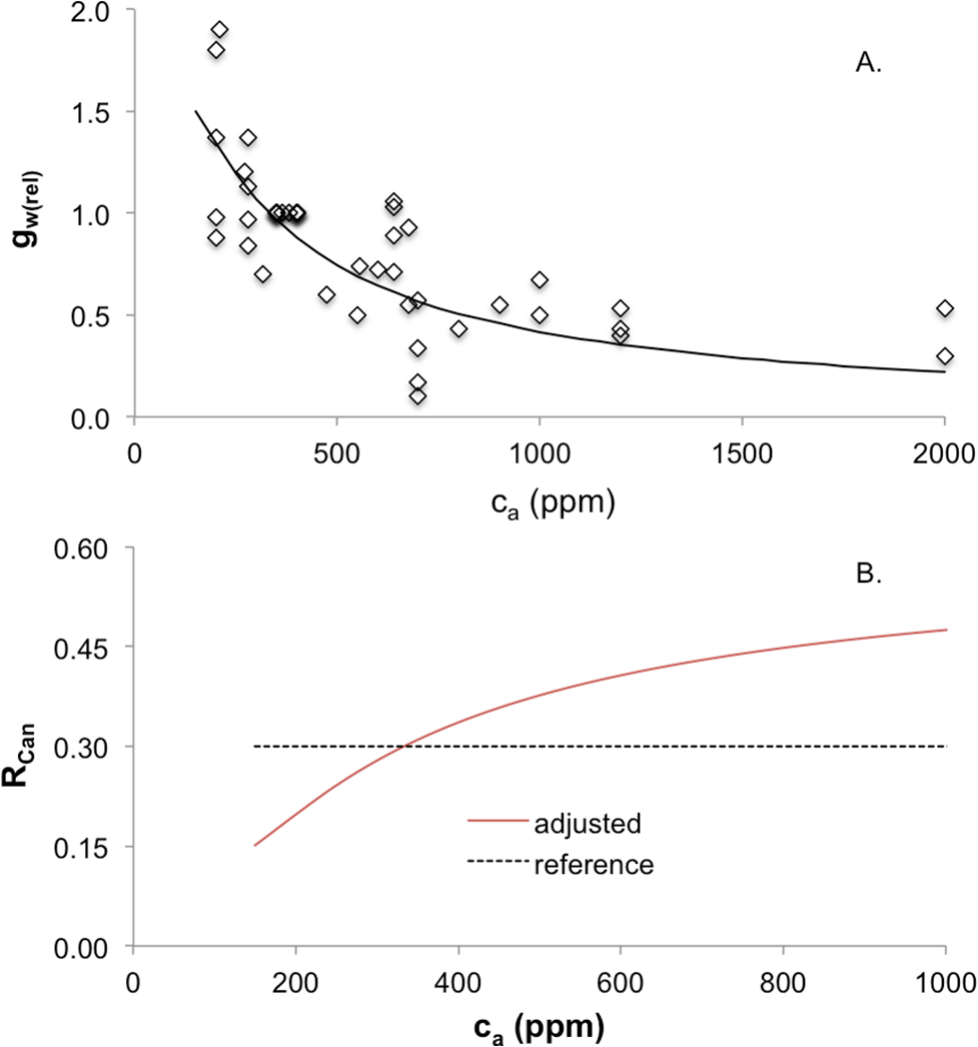
Relationships between atmospheric CO_2_ concentration (c_a_) and (A) relative stomatal conductance and (B) canopy resistance. (A) Observations of relative stomatal conductance of water vapour (g_w(rel)_) under changing atmospheric CO_2_ concentrations (c_a_; data from [44] re-plotted). The solid line is derived from Eq 2B where c_a(rel)_ = c_a_/c_a0_, c_a0_ is 360 ppm, and An_(rel)_ is obtained from Eq 2A. (B) An example of the calculation of adjusted canopy resistance (R_Can,adj_) as a function of c_a_, using a reference canopy resistance (R_Can,ref_) value of 0.3.

Higher atmospheric CO_2_ concentrations (c_a_) are associated with lower stomatal water vapour conductance [40, 41] and greater water use efficiency [42, 43]. These processes are represented in ForWaDy using a function that modifies canopy resistance in each species in relation to projected changes in c_a_ [44]. The functional curve is derived from the physiological relationship between relative net assimilation rate and changes in c_a_ relative to a reference atmospheric CO_2_ concentration (Eq 2A). The impact of c_a_ on stomatal conductance is given in Eq 2B [44], and stomatal conductance in relation to canopy resistance in Eq 3 (see Fig 3B, for an example).

An(rel)=[(ca-r')*(ca0+2r')]/[(ca+2r')*(ca0-r')] (2A)

where:

A_n(rel)_ = relative net assimilation rate

c_a_ = current atmospheric CO_2_ concentration (ppm)

c_a0_ = reference atmospheric CO_2_ concentration (determined from reference climate data, ppm)

r’ = CO_2_ compensation point = 40 ppm

and the relative stomatal conductance for water vapour (g_w(rel)_; dimensionless) is

$${\text{g}}_{\text{w}(\text{rel})}={\text{A}}_{\text{n}(\text{rel})}\;/\;{\text{c}}_{\text{a}(\text{rel})}$$ (2B)

where:

c_a(rel)_ = relative atmospheric CO_2_ conc = c_a_/c_a0_ (dimensionless)

Adjusted canopy resistance (R_Can,adj_; dimensionless) is

$${\text{R}}_{\text{Can},\;\text{adj}}={\text{R}}_{\text{Can},\;\text{ref}}+{\text{R}}_{\text{Can},\;\text{ref}}\;*\;(1-{\text{g}}_{\text{w}(\text{rel})})$$ (3)

where:

R_Can,ref_ = reference canopy resistance (dimensionless)

Many models incorporate a representation of the direct effect of CO_2_ fertilization on tree growth [45] based on evidence from short-term studies [46, 47]. There is little evidence, however, supporting the long-term impacts of CO_2_ fertilization on forest growth [48, 49]. As such, the FORECAST Climate model does not presently include this as a control on growth.

#### 2.1.4. Linkage of FORECAST and ForWaDy

**Fig 4:**
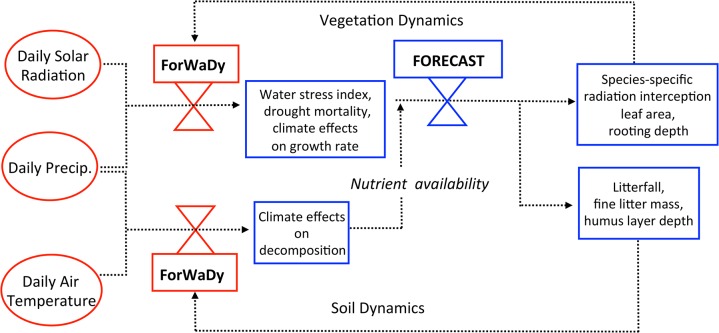
A schematic diagram illustrating feedback loops and data transfer pathways between FORECAST and ForWaDy.

The ForWaDy—FORECAST linkage couples forest water availability directly with ecosystem process simulation. Furthermore, this linkage is dynamic in that the respective functions from each model are continuously updated in response to the iterative sharing of information encoded within a series of feedback loops (see Fig 4). One aspect of this linkage is reconciling FORECAST’s annual time step with the daily time step used in ForWaDy, the latter of which is required to properly capture short-term moisture limitations. This was accomplished by aggregating the daily ForWaDy output into an annual index that was then used as input to FORECAST (Fig 4). FORECAST, in turn, supplies input to ForWaDy at the beginning of each growing season with respect to vegetation and forest floor conditions (Fig 4).

#### 2.1.5. Accounting for climate impacts on productivity

The impact of climate on tree and plant growth in FORECAST Climate is focused primarily on the relationships between growth rate, temperature and water stress. These relationships are represented using species-specific, curvilinear response functions simulated on a daily time step. The net daily growth response index of species i on day d (GRIDay,i,d) is derived as the product of two indices, calculated as temperature and water stress response functions:

$${\text{GRI}}_{\text{Day}\;\text{i},\text{d}}={\text{T}}_{\text{Growth},\;\text{i},\text{d}}\;*\;{\text{S}}_{\text{Growth},\;\text{i},\text{d}}$$ (4)

where:

T_Growth,i,d_ = The temperature response index (range 0–1; dimensionless) for species *i* on day *d*. Specified in the model as a user-defined graphical function of daily mean air temperature (see Section 2.2.3).

S_Growth,i,d_ = The water stress response index (range 0–1; dimensionless) for species *i* on day *d*. Specified in the model as a user-defined graphical function of the daily mean water stress index (see Section 2.2.3).

The temperature response index is designed to encapsulate the complex physiological growth processes governing tree and understory response to variations in mean daily temperature. It is intended to represent net effect of variations in temperature on primary productivity (NPP) by accounting for the impact of temperature on both photosynthesis and respiration. Data for calibrating the index can be derived from greenhouse studies and/or *in situ* measurements of seasonal primary productivity (eddy covariance, for example; see [50]).

The water stress response index is calculated from TDI (see section 2.1.2). The daily TDI value represents the degree to which a species was able to meet its energy-driven transpiration demands (a higher TDI value indicates greater moisture stress). Plants typically respond to water stress by closing stomata to conserve water, leading to an associated reduction in photosynthetic production (see [51]). A negative relationship between S_Growth_ and TDI is therefore expected.

The net daily growth response index is summed to calculate an annual growth response index for year, *y* (GRI_Year,i,y_; Eq 5).

$${\text{GRI}}_{\text{Year},\;\text{i},\text{y}}\sum_{d=1}^{365}{\text{GRI}}_{\text{Day},\text{i},\text{d}}$$ (5)

This index is used as a weighting factor that reflects the ‘quality’ of a given climate year relative to an expected growth rate (further details below).

The climate response functions within FORECAST Climate are calibrated using historical daily climate data. A ‘reference’ daily climate series must be compiled with data derived from a climate station near the forest area to be modeled, and should span a minimum 20-year time period. A summary of the climate data required is provided in Table 1.

Using the reference data set, a calibration run is conducted from which the annual growth response index is calculated (Eq 5). By averaging the annual values for the length of the reference climate period, a normalized growth response index is derived for each species (Eq 6) that reflects growth conditions in an average historical climate year. This index is used in subsequent calculations.

$${\text{NGRI}}_{\;\text{i}}=(\sum_{y=1}^{n}{\text{GRI}}_{\text{Year},\;\text{i},\text{y}})/n$$ (6)

where:

NGRI_,i_ is the normalized growth rate index for species *i* derived from the reference climate data set (dimensionless)

GRI _Year,i,y_ is the annual growth response index for species *i* (Eq 5).

n = the number of years in the historical climate data set

Simulation of growth response to climate in FORECAST Climate is achieved by modifying base growth rates (as determined in the underlying FORECAST model–section 2.1.1) according to the climate relationships described in section 2.1.4. A climate-limited growth rate is calculated by multiplying the base growth rate (Eq 7A) by a climate response factor (Eq 7B).

$${\text{CGR}}_{\;\text{i},\text{y}}\;=\;({\text{BGR}}_{\;\text{i},\text{y}}\;*\;{\text{CRF}}_{\;\text{i},\text{y}})$$ (7A)

$${\text{CRF}}_{\;\text{i},\text{y}}\;=\;({\text{GRI}}_{\text{Year},\;\text{i},\text{y}}-{\text{NGRI}}_{\text{i}})\;/\;{\text{NGRI}}_{\text{i}}$$ (7B)

where:

CGR_,i,y_ is the climate-limited growth rate for species *i* in year *y* (tons ha^-1^),

BGR_,i,y_ = the base growth rate for species *i* and year *y* determined in FORECAST as the light and nutrient-limited growth rate (tons ha^-1^),

CRF_,i,y_ is the climate response factor for species *i* in year *y* (dimensionless), and GRIyear_i,y_ and NGRI_i_ are as defined in Eqs 5 and 6, respectively.

#### 2.1.6. Climate and decomposition rates

The decomposition of dead organic matter in FORECAST is represented based upon user-defined mass loss rates associated with litter quality and derived from field incubation experiments. A maximum of 40 dead organic matter types may be defined and simulated within the model [26]. In FORECAST Climate, decomposition rates are modified by soil moisture content and air temperature with the calculation of a daily decomposition response index (Eq 8). The model does not include any representation of the potential effects of mixing different litter types on mass loss rates.

$${\text{DRI}}_{\text{Day}\;\text{l},\text{d}}\;=\;{\text{T}}_{\text{Decomp},\;\text{d}}\;*\;{\text{M}}_{\text{Decomp},\;\text{l},\text{d}}$$ (8)

where:

DRI_Day,l,d_ = daily decomposition response index (range 0–1; dimensionless) for soil layer *l* on day *d*

**Fig 5:**
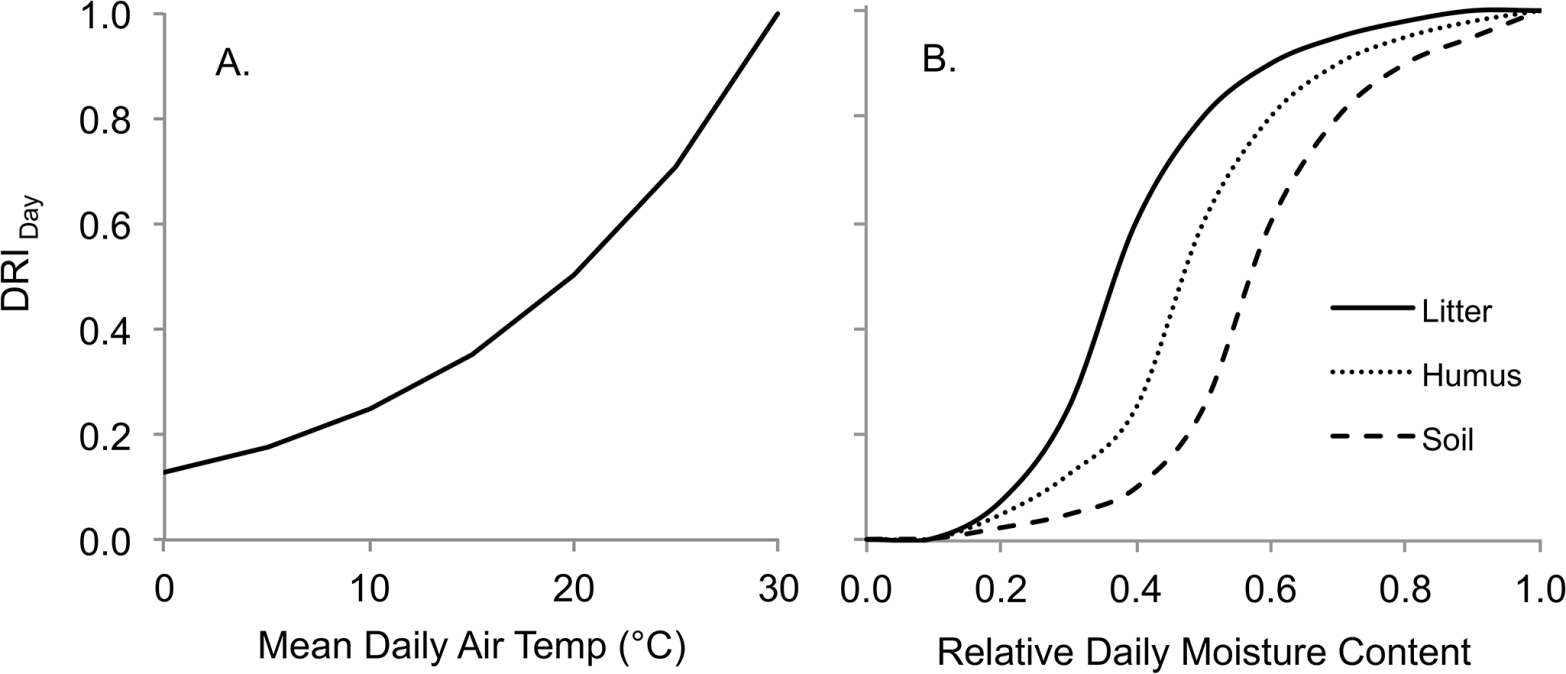
Daily decomposition index in relation to (A) daily air temperature, and (B) relative daily moisture content. (A) The realtionship with mean daily air temperature is based upon a Q_10_ function where Q_10_ = 2. (B) Moisture content relationships are shown for litter, humus, and mineral soil (see text for details).

T_Decomp,d_ = Temperature decomposition response (range 0–1; dimensionless). Specified in the model as a user-defined graphical function of daily mean air temperature (see Fig 5A).

M_Decomp,l,d_ = Moisture decomposition response (range 0–1; dimensionless). Specified in the model as a user-defined graphical function of daily moisture content (see Fig 5B).

Assuming moisture is not a limiting factor, the decomposition rate of dead organic matter is generally faster in warmer environments [52, 53]. The default temperature response function is shown in Fig 5A, and is based upon a Q_10_ factor of 2 [52, 54]. The moisture response functions for litter, humus, and soil, are shown in Fig 5B.

The climate impact on decomposition rates is represented using the same procedure as the growth response index calculation (section 2.1.5). First, a normalized decomposition response index (NDRI_*l*_) is calculated for each soil layer *l* using the reference climate series:

$${\text{NDRI}}_{\;\text{l}}\;=\;(\sum_{y=1}^{n}{\text{DRI}}_{\text{Year},\;\text{l},\text{y}})/n$$ (9)

where:

NDRI_,l_ is the normalized decomposition response index for layer *l* derived from the climate calibration data (dimensionless)

DRI _Year,l,y_ is the summed annual decomposition response index for each soil layer *l*

n = the number of years in the reference climate data set

A climate decomposition factor (CDF_x,y_; Eq 10A) is then calculated as the relative difference between the current and normalized decomposition response indices (Eq 9). Climate feedback is represented by a climate decomposition rate index (CDR_,l,y_) for each soil layer *l* and year *y* (Eq 10B).

$${\text{CDF}}_{\;\text{x},\text{y}}\;=({\text{DRI}}_{\text{Year},\;\text{l},\text{y}}-{\text{NDRI}}_{\;\text{l}})\;/{\text{NDRI}}_{\;\text{l}}$$ (10A)

$${\text{CDR}}_{\;\text{x},\text{y}}\;={\text{BDR}}_{\;\text{x},\text{y}}\;*\;{\text{CDF}}_{\;\text{x},\text{y}}$$ (10B)

where:

CDF_x,y_ is the climate decomposition factor for litter type *x* in year *y* (dimensionless)

DRI_Year,l,y_ and NDRI,_l_, are as defined above (Eq 9),

CDR_,x,y_ is the expected climate decomposition rate for litter type *x* and year *y* (tons ha^-1^)

BDR_,x,y_ is the base decomposition rate for litter type *x* in year *y* determined as a function of litter quality (tons ha^-1^)

#### 2.1.7. Representation of drought-related mortality

Extended periods of drought can lead to plant mortality, either directly or by increasing vulnerability to biotic disturbance agents [5, 55]. FORECAST Climate includes a drought mortality function in which a two-year running-average TDI is used to predict annual mortality. The two-year running average reflects the negative impact of consecutive years of drought (see, for example, [56]). The drought mortality relationship is specified in the model as a user-defined graphical function of species-specific, two-year running average TDI (see Section 2.2.3).

### 2.2. Evaluating model performance using tree-ring analysis

FORECAST Climate was calibrated for a montane forest in south central British Columbia. The model was used to predict annual sapwood production (a proxy measure of tree ring growth), which was then compared against measured 30-year ring chronologies.

#### 2.2.1. Study area

**Fig 6:**
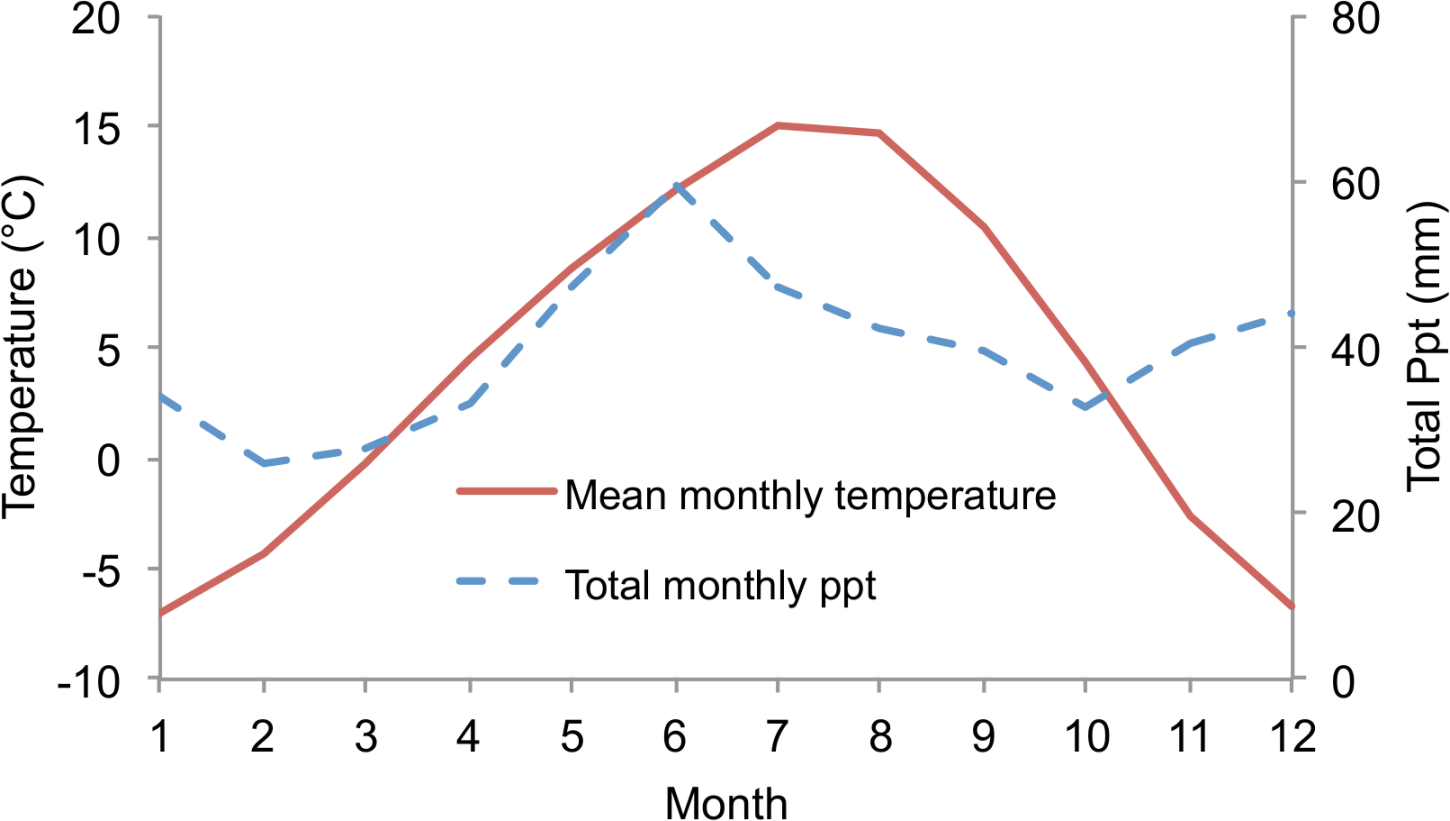
Average monthly temperature and precipitation from the Red Lake climate station (1975–2004).

The study area was located in south central British Columbia, Canada near the city of Kamloops (50°40'00" N, 120°20'00" W), and is classified as a dry cool subzone of the Interior Douglas-fir biogeoclimatic zone [57]. The site, located in crown-owned forest land, is dominated by stands of interior Douglas-fir (*Pseudotsuga mensziesii* var. *glauca*) and lodgepole pine (*Pinus contorta*). Theses species are common in montane regions in the southern interior of British Columbia, ranging from 900 to 1400m in elevation. The closest local climate station with a complete climate record (Red Lake: Elevation = 1162m, 50°56’06” N, 120°48’00” W) was selected from which 30 years of daily data (spanning the years 1975–2004) were obtained. Average monthly temperature and precipitation for Red Lake are shown in Fig 6.

#### 2.2.2. Tree-ring analysis

A total of 5 plots were randomly selected from a series of candidate stands identified using forest inventory data from the study site. Plots were split with 3 Douglas-fir and 2 lodgepole pine-dominated stands. Stands with clear signs of fire scars or damage from biotic agents that could have masked a climate signal were avoided. Plot centers were established using a variable radius to define plot area such that a total of 20 dominant and/or co-dominant trees were identified within the plot area. Species, elevation, slope, aspect, and slope position were recorded for each plot. The stands ranged in age from 36 to 150 years (Table 2).

**Table 2:** Characteristics of sample plot locations from which trees were harvested for tree-ring analysis and chronology statistics.

Plot ID	Species	AverageAge (y)	Elevation (m)	Slope(%)	Aspect (deg.)	Slope Position	Mean sensitivity	Serial correlation
4	Fd	54	1250	25	295	mid	0.288	0.836
9	Fd	67	978	20	50	mid	0.275	0.812
14	Fd	36	1125	18	64	mid	0.244	0.812
34	Pl	60	1300	8	26	low	0.124	0.946
71	Pl	150	1356	2	322	flat	0.159	0.846

A total of 20 trees per plot were sampled. Fd = Douglas fir, Pl = lodgepole pine.

Within each plot, a total of 20 dominant or co-dominant trees were cut down and a slice (cookie) approximately 4 cm thick removed from the base of each stem, within 20 cm of the soil surface. The cookies were prepared for analysis in the Tree-Ring Laboratory at the University of Northern British Columbia, using standard procedures [58]. Annual tree ring width was measured to the nearest 0.01mm using a Windendro system. The ARSTAN software [59] was used to prepare and cross-date the final chronologies. All series were de-trended by fitting a spline of 50% frequency response across 67% of the series length to remove the low frequency age trend. Slight modifications were made to spline stiffness for two of the series to improve their fit to the data. De-trended data were used to isolate the impacts of year-to-year climate variability on relative tree productivity. Chronologies for each plot were constructed using the final ARSTAN tree ring index. Each chronology was restricted to the 1975–2004 period and normalized by dividing by the average ring index. Typically, tree rings are compared against two consecutive years of climate data (current and previous year) [23, 60]. This is because early season wood growth is partly a function of climate suitability in the previous year and its impact on carbohydrate reserves [61].

#### 2.2.3. FORECAST Climate calibration

Detailed descriptions of FORECAST calibration data requirements are provided in [26] and [62]. The base FORECAST model was calibrated with an existing data set from the Interior Douglas-fir dry cool subzone [63]. The data set includes both Douglas-fir and lodgepole pine, and two minor vegetation functional groups (grasses and shrubs) common to the area. Calibration of the climate response functions in FORECAST Climate is described in the following sections.

The hydrological submodel requires data describing characteristics of the soil profile (Table 3) and characteristics of simulated trees and minor vegetation with respect to the calculation of daily evapotranspiration and water stress (Table 4). Average soil characteristics were inferred from soil pits dug in each of the plots.

**Table 3:** Parameter values in the hydrological submodel specific to the simulation of plant-available water within soil layers.

Soil layer	Soil Texture Class	Coarse Fragment Content (%)	Soil Depth^1^ (cm)	Field Capacity Moisture Content ^2^
Humus	N/A	0	12.5	0.32
Mineral A	Silt loam	25	40	0.25
Mineral B	Silt loam	25	45	0.25
Mineral C	Silt loam	25	50	0.25

^1^ The starting humus depth is shown, but humus depth can change over time depending on rates production and decomposition.

^2^ Volumetric water content above which drainage occurs.

**Table 4:** Parameter values in the hydrological submodel specific to the simulation of evapotranspiration and water stress for trees and minor vegetation.

Species	Canopy Parameters		Permanent Wilting Point^3^		Maximum Root Depth
	Albedo^1^	Resistance^2^	Humus	Mineral Soil	(cm)
***Trees***					
Douglas-fir	0.12	0.3	0.08	0.12	100
lodgepole pine	0.12	0.24	0.1	0.13	100
***Minor vegetation***					
*Calamagrostis* grass	0.12	0.15	0.09	0.11	75
*Vaccinium* shrub	0.12	0.10	0.10	0.11	75

^1^ Estimated values.

^2^ Reference relative canopy resistance to water loss through stomata (R_Can,i_) [64]. Higher values indicate greater resistance.

^3^ Relative volumetric moisture content (proportion of total volume) at which soil water uptake is suspended [65, 66].

**Fig 7:**
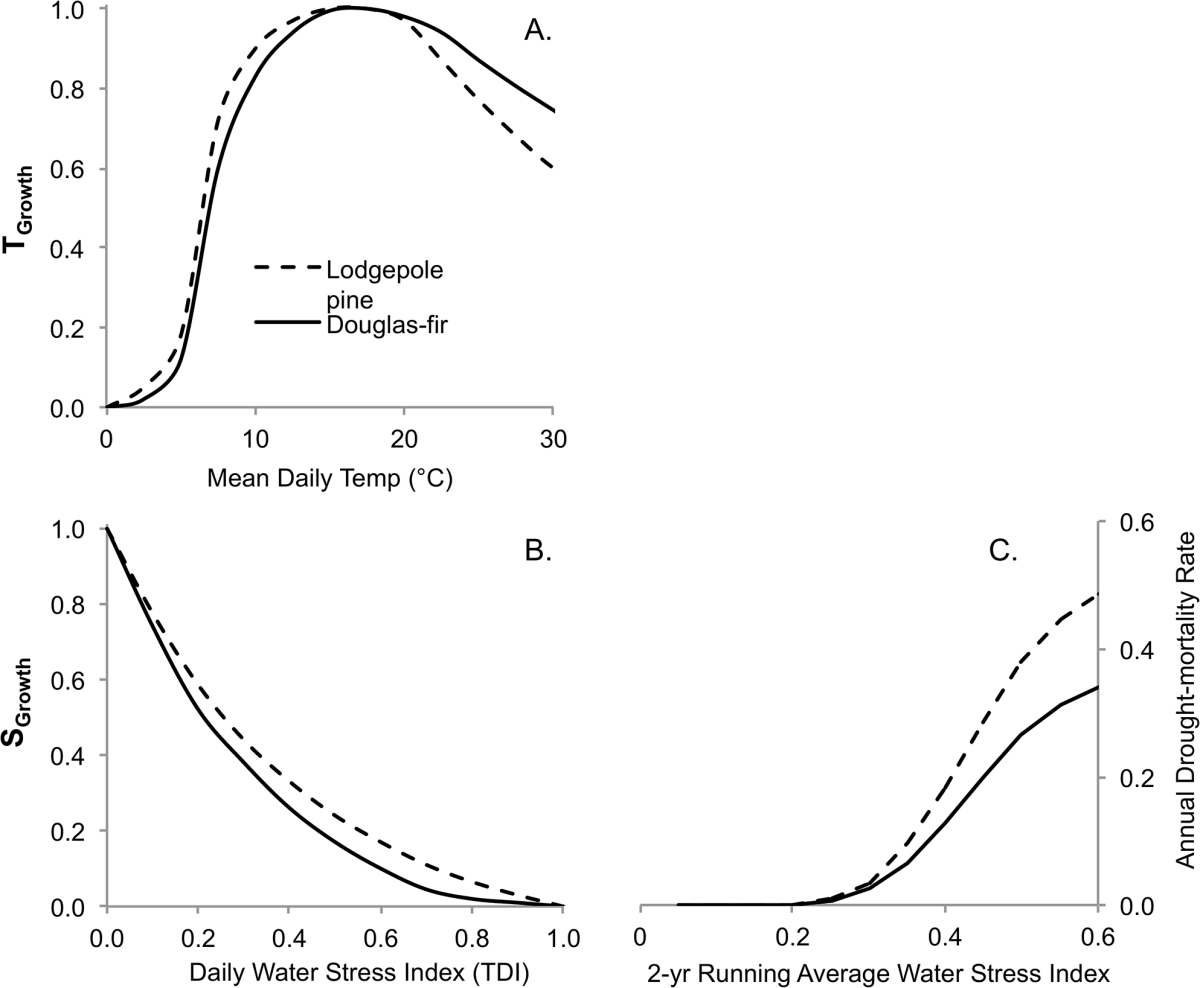
Climate response functions showing the effect of temperature and water stress on growth (A,B) and mortality (C). (A) Relationships between mean daily temperature and the T_growth_ parameter, and (B) daily water stress and S_growth_ parameter used in Eq 4. (C) The drought-related mortality rate as a function of the 2-year running average water stress index.

Calibration of the climate response functions was completed as follows. Daily temperature and water-stress growth response curves were developed for Douglas-fir [67,68] and lodgepole pine [69, 70] based upon literature reviews of climate-growth relationships (Fig 7A and 7B). The sharp increase in growth beginning around 5°C for both species is indicative of the heat sum required to break dormancy. Each curve varies with respect to the optimal temperature range for growth and the relative rate of decline beyond the optimum, depending on a plant’s tolerance characteristics. The decline at higher temperatures represents the increase in respiration associated with elevated temperatures [50].

A relative drought mortality curve was developed for each tree species based on its ecological characteristics within the subzone (Fig 7C). Douglas-fir was assumed to be slightly more tolerant of drought stress relative to lodgepole pine. Each species-specific mortality function was evaluated using provincial forest health survey data (not shown) to verify that it produced realistic mortality projections when the model was run with the historical reference climate data.

#### 2.2.4. Growth simulation using historical climate data

To evaluate the capability of FORECAST Climate to reproduce the measured, climate-related annual variation in tree ring growth, the model was run using the historical climate data from the period of 1975–2004. The 30-year climate data set was cycled 5 times to generate 150 years of daily data. Model projections of annual sapwood production were subsequently compared against the normalized tree ring indices for the corresponding climate years using regression analysis. However, because annual ring growth is fueled both by carbohydrates stored from the previous year and the current year’s production, each ring index was compared against a normalized average of the previous and current year’s simulated sapwood production.

### 2.3. Simulation of long-term climate change impacts

#### 2.3.1. Development of climate change scenarios

**Fig 8:**
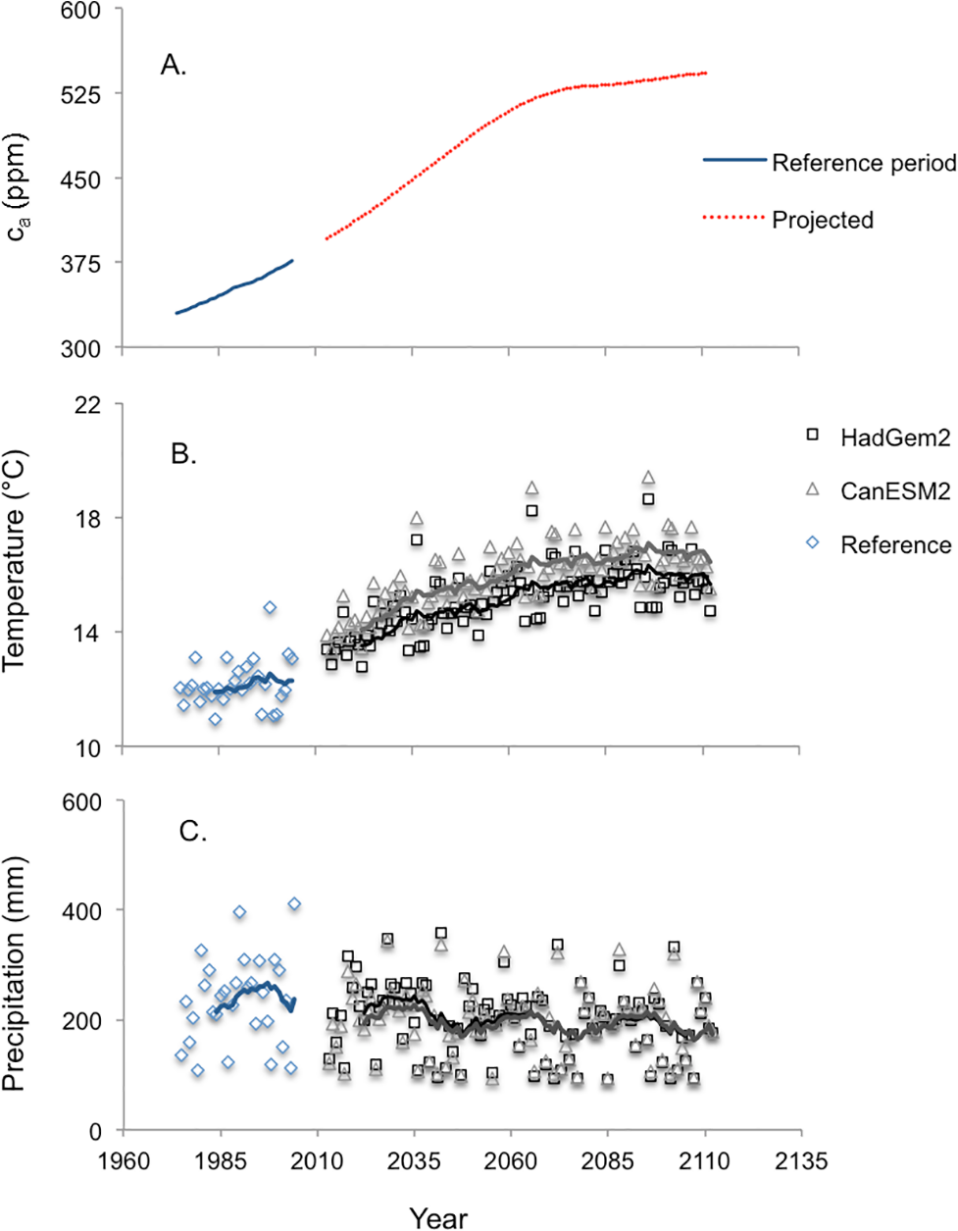
Reference climate data and projected atmospheric [CO_2_] (A), growing season temperature (B), and precipitation (C). (A) Projected increase in atmospheric [CO_2_] (c_a_) for the RCP 4.5 emissions scenario, (B) projected growing season (MAY–SEP) mean daily air temperature, and (C) projected total growing season precipitation for the next 100 years based on two downscaled climate change projections (see text). Lines represent the 10-year moving average for each series in panels B and C.

To illustrate the potential of FORECAST Climate to simulate the impacts of climate on the growth and development of forests within the study area, climate change projections were derived from two established general circulation models (GCMs) included as part of the Intergovernmental Panel on Climate Change AR5 analysis [31], HadGem2 (www.enes.org/models/earthsystem-models/metoffice-hadley-centre/hadgem2-es) and CanESM2 (www.cccma.ec.gc.ca/data/cgcm4/CanESM2/rcp45/index.shtml). A medium range emissions pathway was selected for the two models (Fig 8A). It is based on a representative CO_2_ concentration pathway that generates a radiative forcing of 4.5 Wm^-2^ (RCP 4.5, [71]). These models and scenario were selected to be representative of the middle of the range of potential climate change scenarios. It should be noted that other scenarios could generate different results. Atmospheric CO_2_ concentration for the reference climate scenario was constrained to 2005 values (380 ppm). The regional monthly output from the GCMs for the 2020s, 2050s and 2080s was downscaled to daily data using a direct approach based upon the Red Lake daily climate data series from the years 1975–2004. The resulting data set spanned a 100-year period (2012–2111). Projected changes in mean growing season temperature and precipitation are shown in Fig 8B and 8C. Mean growing season temperature is predicted to increase from approximately 12.5°C in 2013 to 16–17°C by 2111. In contrast, both GCMs predicted only a very gradual declining trend in growing season precipitation during the same period. It should be noted that the interannual variability in temperature and precipitation characteristic of the base reference data is retained as a result of the direct downscaling process.

#### 2.3.2. Model application for climate change simulation

FORECAST Climate was used to simulate the long-term impact of climate change on stand growth and development for both Douglas-fir-dominated and lodgepole pine-dominated stand types. Growth in newly planted (age = 1 year) and established stands (starting age = 60 years) was simulated for a 100-year period. In the newly planted stands, the climate began to change immediately (see Fig 8). For established stands, however, the 30-year historical climate data were copied and appended to create 60 years of data before the climate change period began at age 61. A total of eight runs were completed (2 climate scenarios * 2 species * 2 age classes).

## Results

### 3.1. Model evaluation against tree ring data

**Fig 9:**
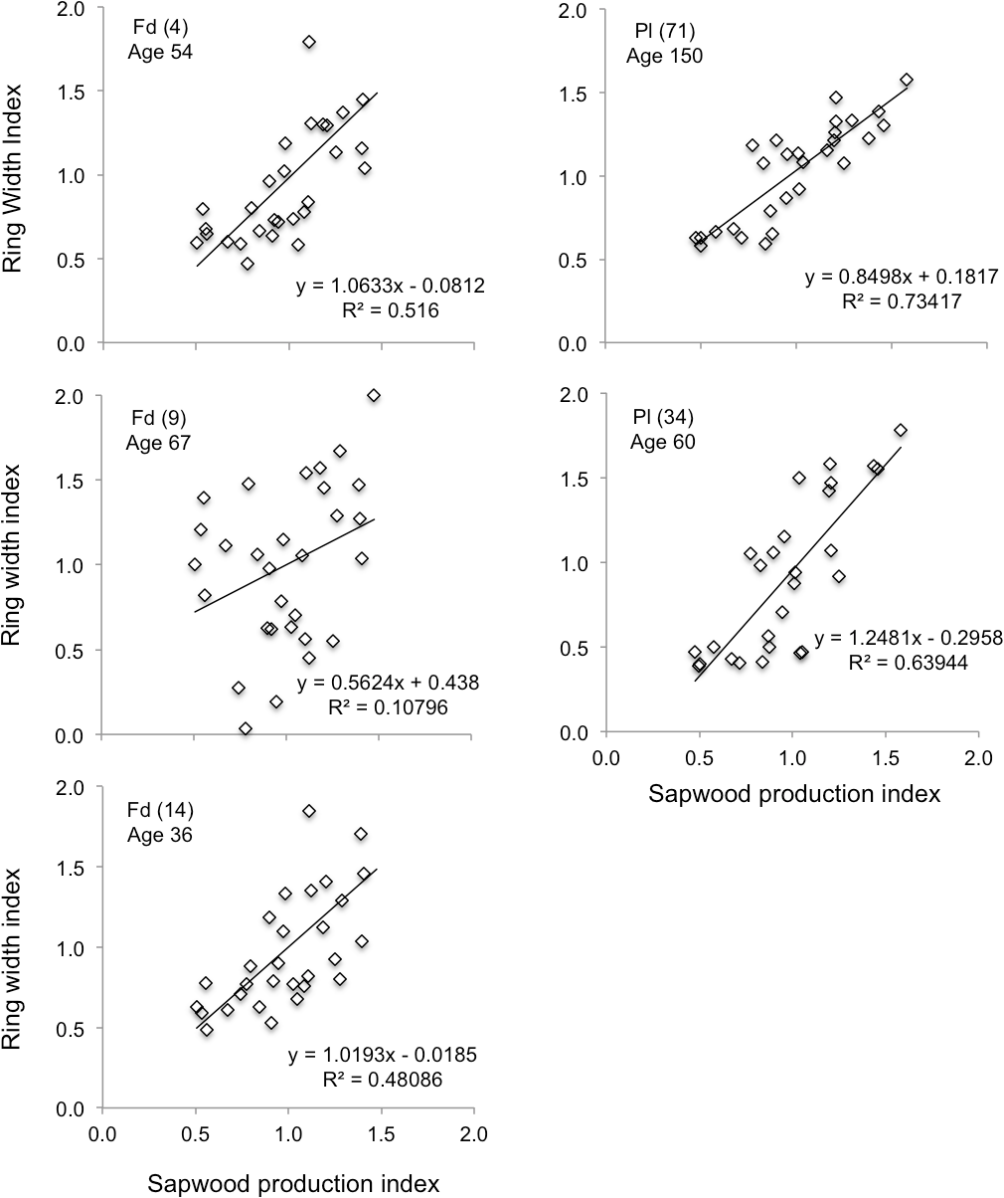
Comparison of the simulated sapwood production index with the measured ring width index. Data are shown for Douglas-fir (Fd) and lodgepole pine (Pl) plots for the 30-year climate reference period. Average tree age is shown for each plot.

Sapwood production predictions were concordant with 48% to 73% of the variation in measured annual ring widths in 4 of the 5 plots (Fig 9). One Douglas-fir plot (Plot #9) showed no significant correlation with the other plots suggesting that annual variation in tree growth in this plots was likely a consequence of factors other than climate. This plot was therefore excluded from subsequent analysis. The average slopes of the regression lines were 1.04 and 1.05, for the remaining Douglas-fir (#4 & #14) and pine (#71 & #34) plots, respectively.

### 3.2. Climate change impacts on tree growth

**Fig 10:**
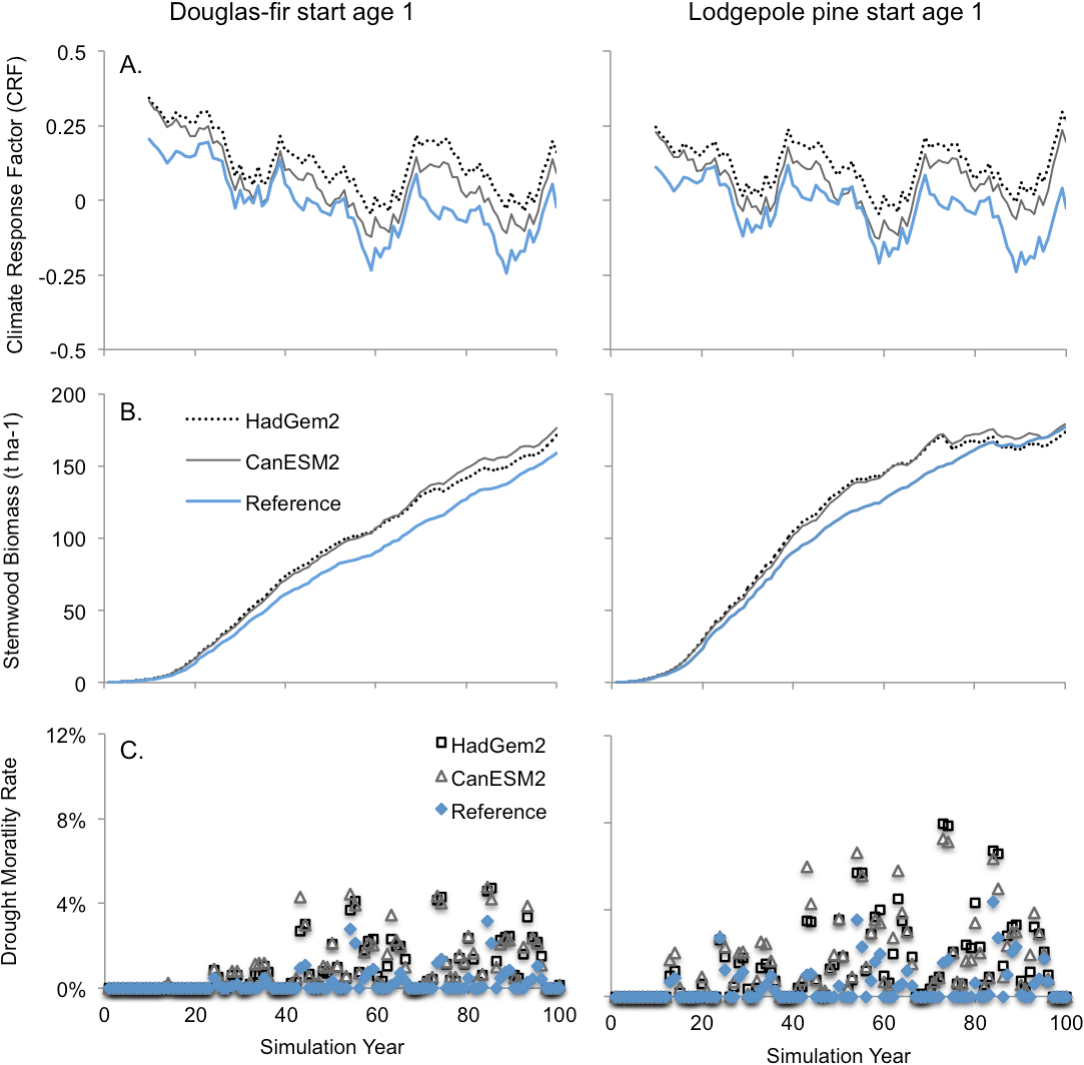
Impact of climate change on: (A) growth response (CRF), (B) stemwood biomass, and (C) drought-related mortality. Simulations results for newly planted stands of Douglas-fir are shown on the left and for lodgepole pine on the right. Climate change simulation was initiated at year 1 of the simulation. Lines represent 10-year running averages of annual values. A CRF value near zero indicates that the simulated climate year was similar to the historical average year.

**Fig 11:**
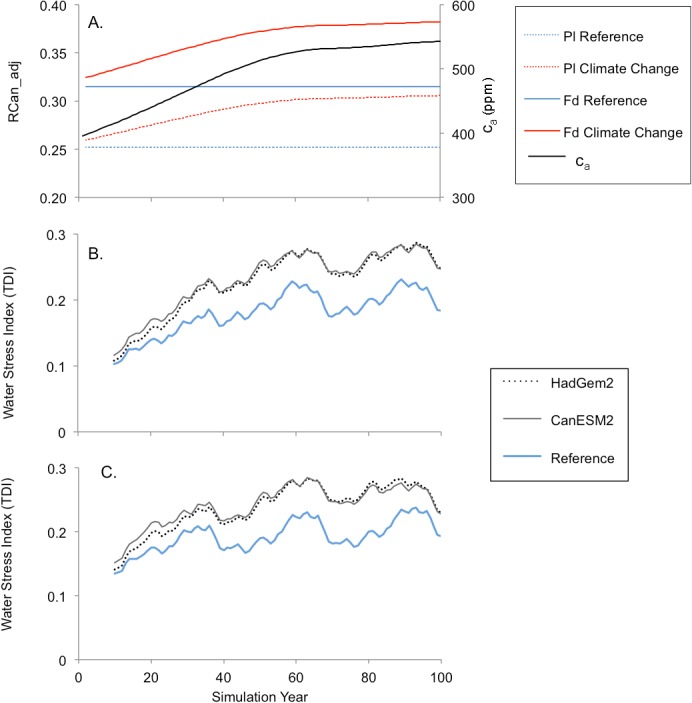
Simulated effect of c_a_ on canopy resistance (A) and of climate change on tree water stress (B-C). (A) Projected atmospheric [CO_2_] (c_a_) for the climate change scenarios is based on the RCP 4.5 emissions scenario while reference scenario levels were constrained at 2005 values (380 ppm). The impact of c_a_ on canopy resistance (R_Can,adj_) is shown for both species and for both climate scenarios (see Eq 3). Lower panels show simulated water stress for newly planted stands of (B) Douglas-fir and (C) lodgepole pine. Water stress results represent the 10-year running averages of annual transpiration deficit index (TDI) values.

Both climate change scenarios had a net positive impact on growth rates for newly planted stands, as indicated by the climate response factor (CRF, Eq 7; Fig 10A). Ten-year running averages show that the CRF values under the historical (reference) climate oscillate around zero, while those for the climate change scenarios are consistently higher. The impact of climate change on stemwood biomass was generally positive for both tree species, except in pine stands 85 years and older (Fig 10B). In Douglas-fir, stemwood biomass was 11 to 17% higher at rotation age (80 years), and 4 to 7% higher in pine. Climate change triggered more drought-related mortality (Fig 10C), which was associated with an increase in both the frequency and intensity of water stress (Fig 11B and 11C). The increase in water stress in the climate change scenarios occurred despite an increase in canopy resistance associated with higher CO_2_ concentrations (Fig 11A).

**Fig 12:**
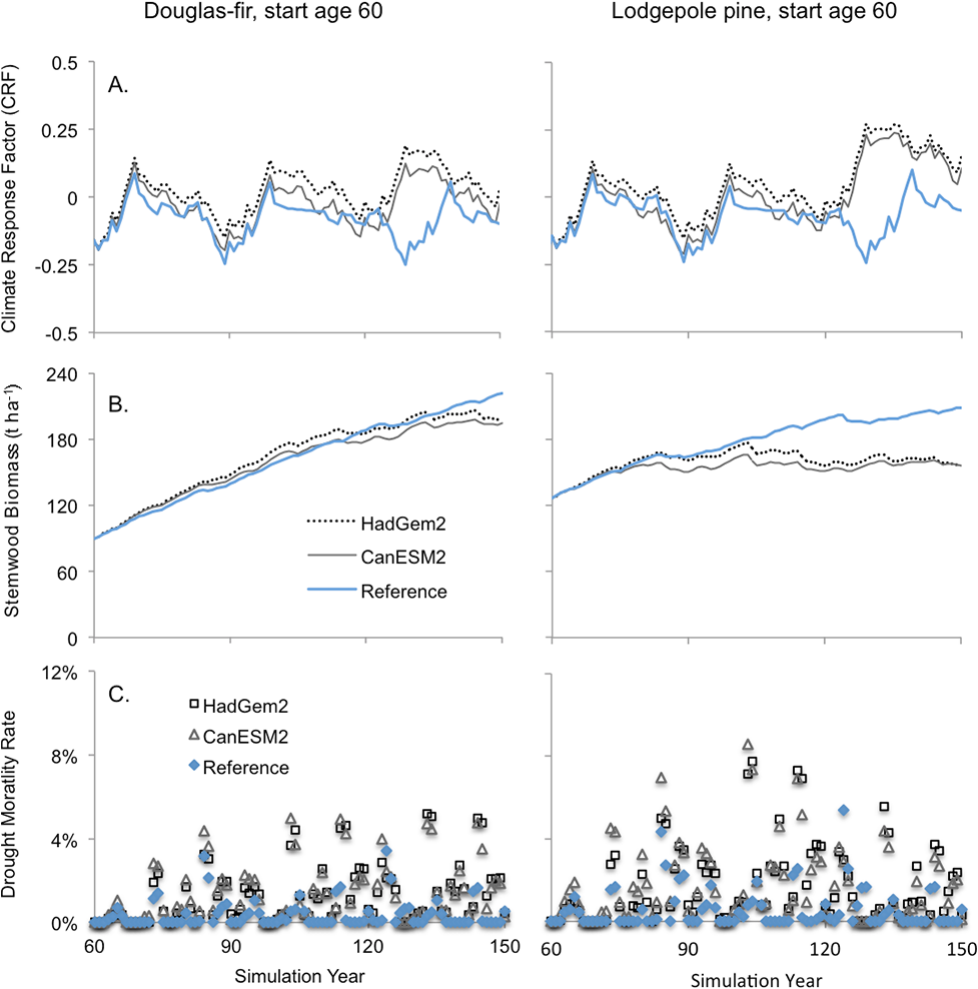
The impact of climate change for on: (A) growth response (CRF), (B) stemwood biomass, and (C) drought-related mortality. Simulation results for established (60 years old) stands of Douglas-fir are shown on the left and for lodgepole pine on the right. Climate change simulation was initiated at year 60 of the simulation. Lines represent 10-year running averages of annual values. A CRF value near zero indicates that the simulated climate year was similar to the historical average year.

In established (60 year-old) stands, the impact of climate change was projected to be positive in terms of the CRF (Fig 12A). This was not the case for stemwood biomass, however. For Douglas-fir, there was little difference among the historical and climate change scenarios, though biomass under the latter was lower when stands were very old (Fig 12B). In pine, however, climate change reduced stemwood biomass early in the simulation. This discrepancy became more pronounced over time. Lower stemwood biomass was associated with elevated drought-related mortality in both species but more so in pine (Fig 12C).

**Fig 13:**
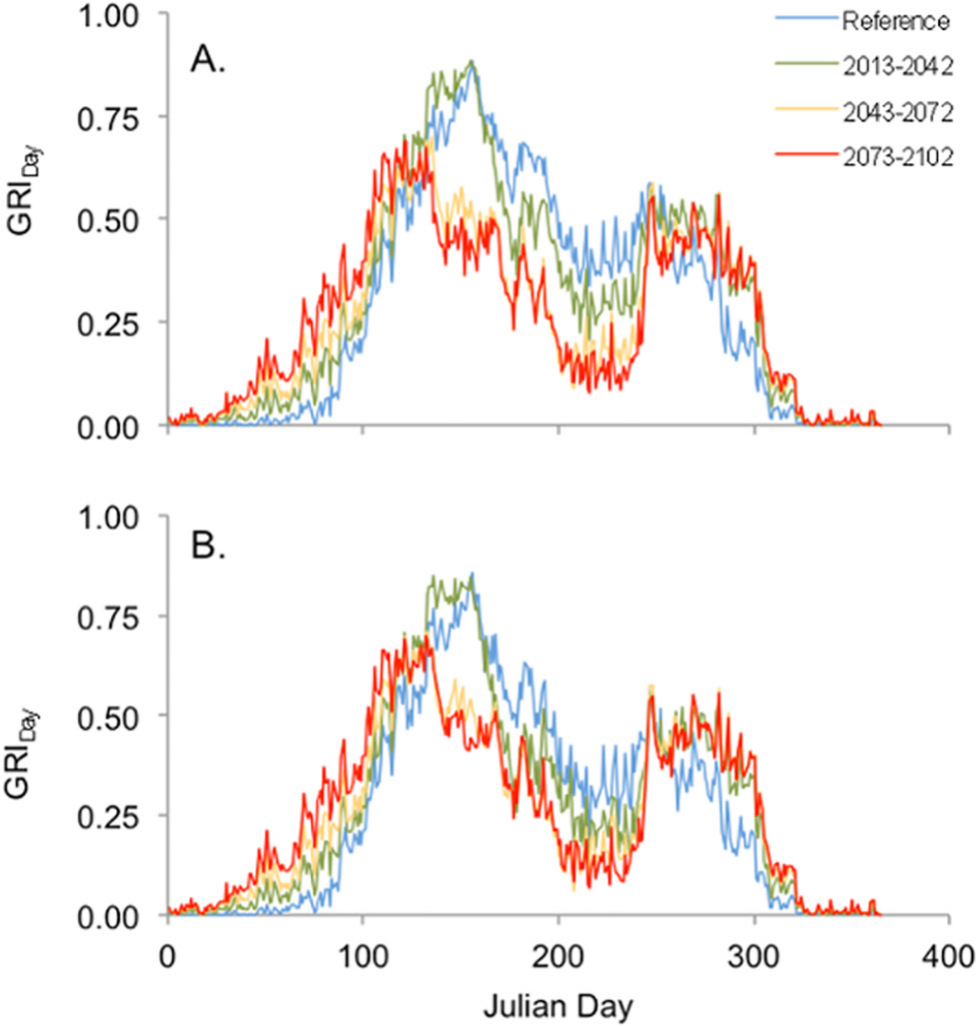
Simulated impact of climate change on GRI_Day_ for Douglas-fir (A) and lodgepole pine (B). Results for mean daily growth response index (GRI_Day_) are shown for newly planted stands and reflect data from the 30-year historical time period (reference), and three sequential 30-year future climate periods (2013–2042, 2043–2072, and 2073–2102) based upon the CanESM2 climate scenario.

Projected impacts in newly planted stands of the reference climate and climate change (CanESM2) scenarios on the daily growth response index are shown for three future climate periods (Fig 13). The daily growth response index (GRI_Day_) is determined as the product of the daily growth response to temperature (T_Growth_) and water stress (S_Growth_) (Eq 4) and is summed for the year to determine the annual growth response index (GRI_Year_). The model predicts a considerable lengthening of the temperature-limited growing season, as approximated by the number of calendar days where the value of T_Growth_ ≥ 0.25 (data not shown). The average length of the temperature-limited growing season for both species increased by 42 days in the period from 2073–2102 relative to the reference period (1975–2004). The increase, however, was also accompanied by higher daily drought stress during the growing season leading to a mid-summer decline in average GRI_Day_ (Fig 13).

### 3.3. Climate change impacts on decomposition

**Fig 14:**
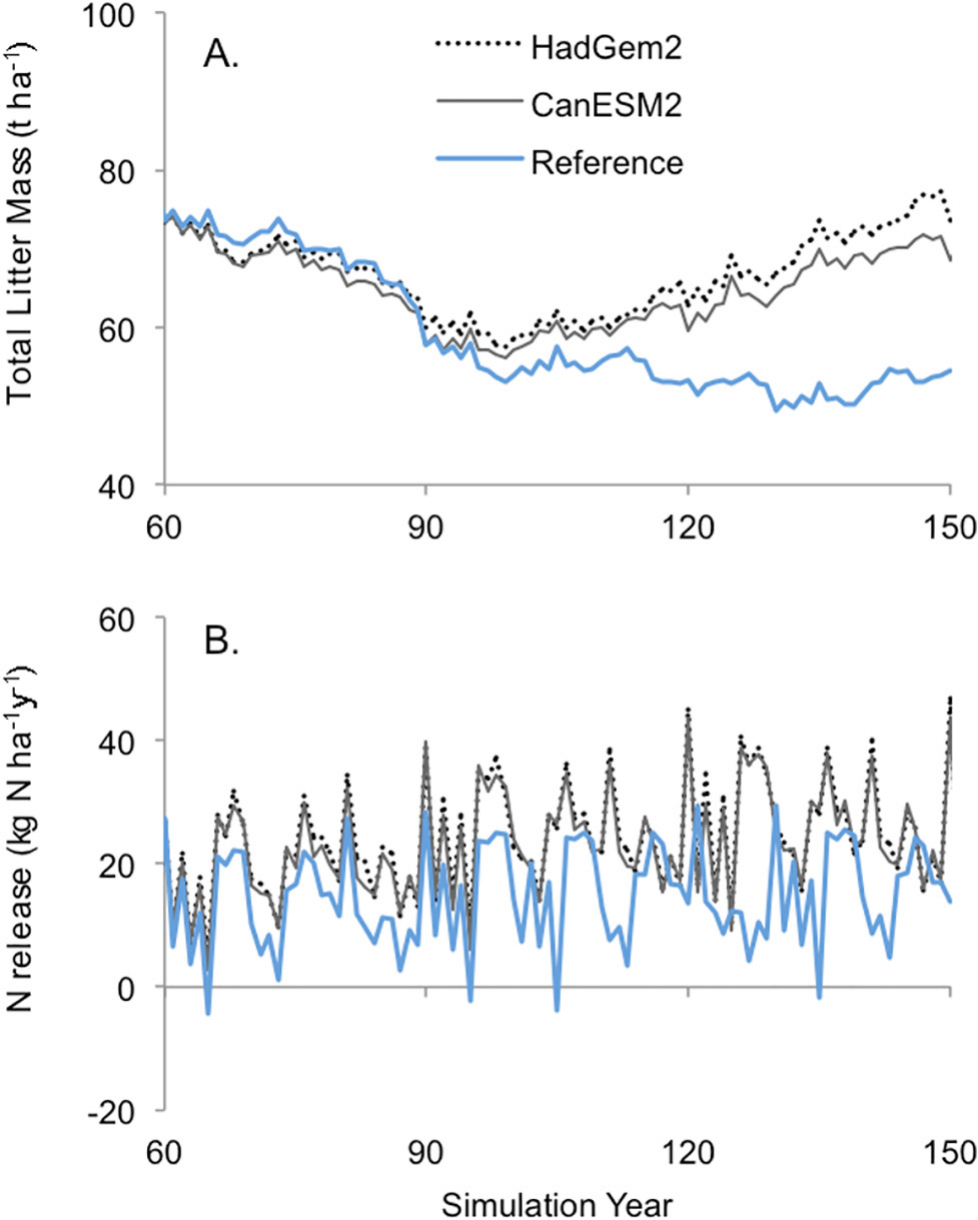
Simulated impact of climate change on (A) total litter mass, and (B) net N release. Results are shown for established (60 years old at the start of the climate change simulation) Douglas-fir stands.

Over the first 30 years of the simulation, climate change led to reduced levels of litter mass due to increased mass loss rates (Fig 14A). Thereafter, total litter mass in the climate change scenarios increased to levels above that of the reference climate. Nitrogen (N) release was consistently higher under both climate change scenarios (Fig 14B).

## Discussion

### 4.1. Model evaluation using tree-ring analysis

A variety of metrics have been useful in establishing the relationship between climate and plant productivity (see [72]). One metric used extensively is dendrochronology, in which changes in ring width are linked to annual variation in climate, particularly temperature and moisture [73]. Dendrochronology thus provides a means of assessing how well a forest model can project the impact of climate on plant performance (i.e., annual ring growth). If a model can successfully reproduce ring chronologies as a function of historical climate conditions, it should be reasonable to use these same relationships to predict the future growth response to a changing climate regime (see, for example, [74, 23, 60, 75]).

Dendrochronological records indicate that, in general, annual tree growth in temperate regions tends to be more sensitive to variations in water stress than temperature [76, 77]. FORECAST Climate performed well in capturing the historical effect of climate on ring width indices in 4 of the 5 measured plots. Model output was consistent with years when measured radial growth rates were reduced due to water stress as well as years in which growth was above average.

The positive impacts of temperature on ring width were captured directly by FORECAST Climate as a lengthening of the growing season, and indirectly through the positive influence of enhanced nitrogen mineralization on site productivity.

One of the challenges in using tree ring data is that climate signals can be obscured by other factors that influence growth, such as abiotic and biotic disturbance agents, and competition for site resources from neighboring trees and/or other vegetation types [73]. In our case, one of the two plots with lower R^2^ values (Douglas-fir, plot #9; see Table 2) displayed unusual patterns whereby ring widths declined to near zero for a three-year period before returning to ‘normal’ levels (data not shown). The other Douglas-fir plots showed no such pattern suggesting that the decline was not climate related. Abrupt disruption and subsequent recovery in tree radial growth is indicative of insect attack. In Douglas-fir that might be from budworm or bark beetle [78]. Regardless of its source, the ring pattern was sufficiently skewed that it effectively obscured the climate signal.

### 4.2. Effects of climate change on productivity and mortality

FORECAST Climate predicted an overall increases in annual growth rates under future climate conditions in both Douglas-fir and lodgepole pine (Figs 10 and 12). An increase in growing season length was the dominant factor in this regard and served to counteract an increase in the frequency and intensity of summer moisture stress, the latter of which represented a downward pressure on growth (Fig 11). Summer moisture stress trended upwards under climate change despite simulated increases in canopy resistance associated with elevated atmospheric CO_2_ concentrations (see Fig 11). [23] also reported an antagonistic effect of growing season length and moisture stress on forest productivity in the boreal forest. [79] found that Douglas-fir growth rates in warm-dry regions correlated well with annual precipitation while growth in populations from cooler and wetter regions was better correlated with temperature and other climatic variables. Summer moisture deficit was the key climatic factor limiting interior Douglas-fir growth rates in montane ecosystems in the northwestern US [77]. In the Cascade Range, Washington, USA, [80] observed that growing season precipitation was the climate variable most strongly correlated with growth rates in mid-elevation lodgepole pine stands, indicating that summer water stress was a key limiting factor. They noted that temperature-regulated growing season length was the most important factor influencing growth rates in high elevation stands.

Higher drought-related mortality was predicted by FORECAST Climate in all climate change scenarios. This resulted in lower biomass production except for newly planted Douglas-fir stands. Established stands were vulnerable to the warmer and drier climate conditions due to a high leaf area. This is because older stands originated and developed under historical climate conditions, which can support a greater leaf area. The latter, however, is poorly adapted to an increasingly water-limited future environment. High evapotranspirational demand in older stands thus triggered widespread mortality during drought and a significant loss of live biomass. Although newly planted stands also incurred elevated mortality, the impact on long-term productivity was relatively low and surviving trees developed canopies with a lower leaf area. Finally, it should be noted that the intensity of predicted water stress declined after a mortality event. This was because mortality reduced stand leaf area (data not shown) resulting in a lower canopy water demand.

Evidence of increased drought-related mortality has been documented in forest ecosystems around the world [5, 81, 14]. Moreover, [82] observed widespread growth decline despite increases in water use efficiency in temperate and boreal forests in a study combining standard dendrochronological techniques with isotopic analysis. The complex physiological response of vegetation to water stress, however, has made it difficult to represent accurately within forest ecosystem models [78]. Prolonged moisture stress increases mortality generally [5, 83] and both hydraulic failure and carbon starvation have been implicated as direct causal factors [84, 51]. Trees suffering from water stress are also more vulnerable to abiotic and biotic disturbance, including fire and insect attack [6, 83]. The water-stress mortality function employed in FORECAST Climate was designed to encapsulate both the direct and indirect aspects of drought-related mortality. It can be parameterized through an iterative process whereby modeled mortality derived using historical reference climate data is adjusted to achieve a reasonable match to measured rates.

It should be noted that the projections of the impact of climate change on long-term growth and productivity in these ecosystems do not include any impacts from extreme climate events nor the effect of climate on population dynamics of natural disturbance agents. Both of these factors have the potential to substantially impact forest productivity as the climate continues to change.

### 4.3. Climate effects on decomposition and nutrient cycling

FORECAST Climate predicted a net increase in mass loss associated with litter decomposition over most years in the climate change scenarios and there was an increase in simulated litter mass over the long-term (Fig 14A). The latter was a result of the higher mortality rates that occurred under climate change relative to historical conditions (see Figs 10C and 12C). Though warmer temperatures had a positive impact on decomposition rates there were periods when litter water content was low, which exerted a downward pressure on rates. Nevertheless, the net increase in decomposition led to an associated increase in N mineralization and thus a positive benefit to tree productivity. [11] provided evidence that soil warming leads to pronounced increases in litter mass loss and net N mineralization rates. In an experimental manipulation of soil warming over 7-years, [85] showed enhanced biomass production relative to controls, which they attributed largely to increased N availability. The representation of such feedback loops in models is thus paramount to improving their capability to capture climate change impacts on forest growth and development.

### 4.4. Hybrid modelling approach

FORECAST Climate employs a series of climate response functions to represent the impact of temperature and moisture stress on plant growth. The benefits of this approach are: 1) it represents a relatively simple and transparent approach to predicting the independent and combined effects of temperature and moisture stress on growth, 2) the shape of the response functions can be easily established and evaluated empirically, 3) it provides a convenient means of summarizing daily responses into annual indices, and 4) is easily calibrated using historical reference climate data. Further potential improvements to the model include a representation of the phenological responses of plants to climate, such as bud break or the onset of dormancy, and frost events.

## Supporting Information

S1 File A detailed description of the ForWaDy model, including key equations, algorithms, and references are provided within. (PDF)
