# Supplementary material for: Application of a Hybrid Forest Growth Model to Evaluate Climate Change Impacts on Productivity, Nutrient Cycling and Mortality in a Montane Forest Ecosystem
Source: PLoS One. 2015 Aug 12;10(8):e0135034. doi: 10.1371/journal.pone.0135034 (PMC4534035; doi:10.1371/journal.pone.0135034)
Supplement: S1 File — (PDF) [file pone.0135034.s001.pdf]

Supporting information for the article:

Application of a hybrid forest growth model to evaluate climate change impacts on productivity, nutrient cycling and mortality in a montane forest ecosystem

Material S1

Brad Seely[1]*, Clive Welham[1] and Kim Scoullar[2]

1. Dept. of Forest Resources Management, University of British Columbia, Vancouver, BC, 2424 Main Mall, Vancouver, BC, V4A6L1, Canada

2. Life Sciences Programming Inc., R.R. #1, Site 5, Comp. 25, 920 Aikins Loop, Naramata, BC V0H 1N0

*Corresponding author

Brad Seely
Phone: 604-880-1593
email:brad.seely@ubc.ca
address:
Dept. of Forest Resources Management
University of British Columbia
2424 Main Mall
Vancouver, BC, V4A6L1
Canada

Clive Welham
email:clive.welham@ubc.ca

Kim Scoullar
email:kscoullar@shaw.ca

1. ForWaDy Description

The ForWaDy (Forest Water Dynamics) model was constructed around the foundation of FORHYM, a general water balance model designed by [2] for the simulation of water fluxes through temperate forest ecosystems. It includes a representation of the vertical flow of water through canopy and soil layer compartments (Fig. S1). Movement of water through each soil layer is regulated by its physical properties that dictate moisture holding capacity, permanent wilting point moisture content, and infiltration rate.

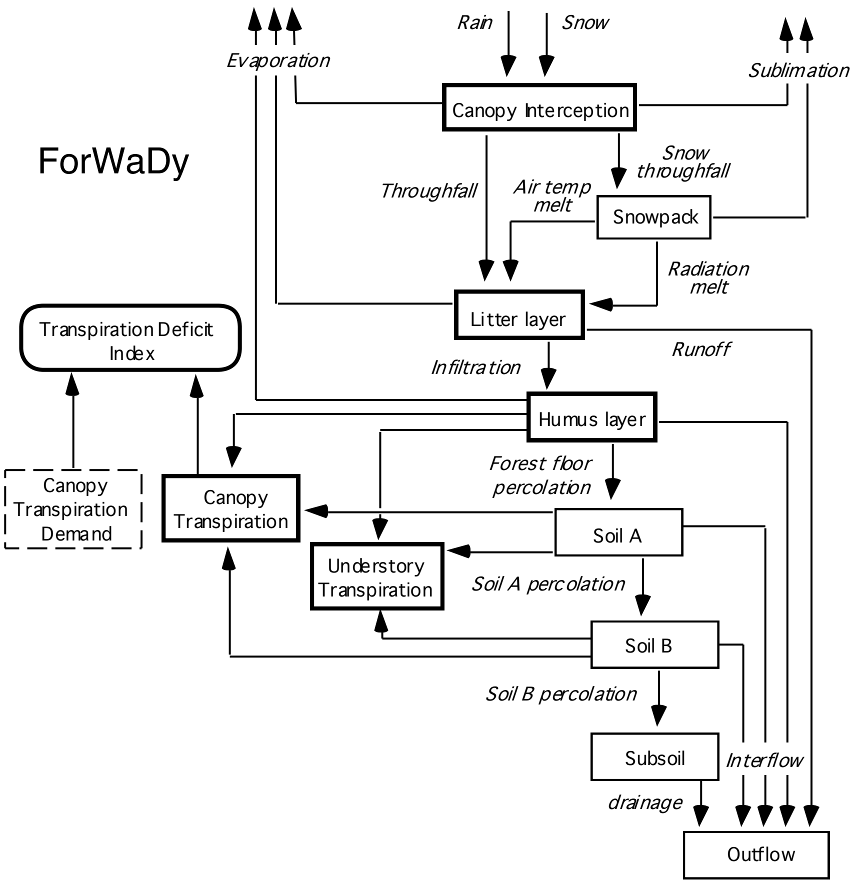

Figure S1. Schematic diagram of the ForWaDy model indicating water flow pathways and storage compartments.

The daily energy available for evapotranspiraton is divided among canopy trees, understory plants and the forest floor based on the proportional interception of incoming solar radiation by each layer adjusted for reflection depending on surface albedos. Subsequently, a passive competition for available soil moisture is simulated through the use of an algorithm that combines species-specific root occupancy information with energy-limited transpiration and evaporation demands. Canopy water stress is determined as a function of energy-limited canopy transpiration demand and soil-limited actual canopy transpiration through the calculation of a cumulative water stress index. This index represents a dynamic measure of tree water stress over a given time period and may be used as a parameter to limit tree growth rates based on light and nutrient availability in models such as FORECAST and FORCEE. The simulation of snowfall and snowpack dynamics in ForWaDy is based on the RHYSSys Snow Model [4].

All equations describing water flow within the model are solved using a simulation dt of 0.25 days. The use of a dt less than one day enables the model to divide the flux of water from a particular soil reservoir among competing outflows.

S1.1. Data Requirements

One of the goals in model development was to produce a model that is portable and suitable for use in forest management; thus, climate and site-specific soil and vegetation data requirements were kept to a minimum. The use of parameters that must be calibrated for each site was also avoided when possible. Data requirements are shown in Table S1.

Table S1. Data requirements for ForWaDy

Climate data (daily)	Vegetation data	Forest floor & soil data
mean, max and min air temperature	seasonal conifer and hardwood LAI	fine litter mass (kg/ha)
solar radiation[1]	seasonal understory % cover	humus layer depth and bulk density
total precipitation	rooting depths for trees	depth of mineral soil layers
snow fraction	rooting depths for understory	soil texture (by layer)
atmospheric [CO_2][2]	canopy resistance and albedo (by	coarse fragment content (by layer)

	species)	

[1] Solar radiation may be estimated from max and min air temperature, elevation, latitude, slope and aspect using published radiation models.

[2] Only annual data required.

S1.2 Radiation interception

Radiation interception in ForWaDy may be estimated from a host model as is the case with FORECAST Climate or it may be done using the default equations within ForWaDy as follows. The energy available for driving evapotranspiration is estimated separately for the canopy, understory, and forest floor based upon the proportion of adjusted total daily solar radiation intercepted by each layer. The interception and extinction of radiation as it passes through the canopy and subsequently through the understory is calculated as a function of LAI using a simple formulation of Beer's Law with an extinction coefficient of 0.4 (Figure S2). Forest floor radiation is calculated as the total remaining radiation following simulated canopy and understory interception. For simplification purposes, the transmittance of net radiation (Sn) is assumed to be equal to that of shortwave radiation [10] following the reflection of a portion of total incident shortwave radiation according to a surface albedo (a) (Eq. S1). The default albedo (a) for the canopy and understory is set at 0.12 which is representative of typical values reported for forests [18, 9] and should be used as a reasonable approximation in the absence of a measured value. The forest floor albedo is determined as a function of solar zenith angle and moisture content according to [22].

$$Sn = Rad * (1 - a) \qquad \text{(S1)}$$

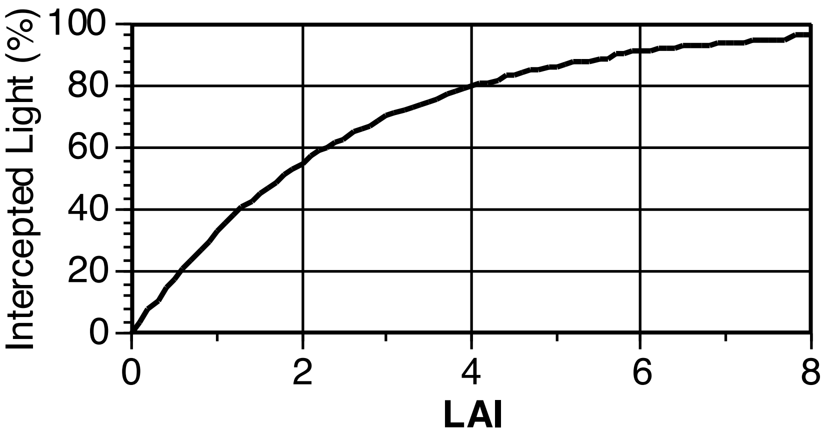

Figure S2. The relationship between leaf area index (LAI) and intercepted light as a percent of total above canopy light according to Beer's Law with an extinction coefficient of 0.4.

S1.3 Energy-limited evapotranspiration

Energy-limited or potential evapotranspiration (PET) is calculated for each layer represented in the model based on the following simplification of the Penman-Monteith equation proposed by [16]:

$$PET = \alpha \frac{s}{s+\gamma}(Sn - G - M)\left(\frac{1}{L}\right) \qquad \text{(S2)}$$

where α is an experimentally determined coefficient, s, γ, and L are respectively, the slope of the saturation vapor pressure curve, the psychrometric constant, and the latent heat of vaporization of water each evaluated at the daily average air temperature and Sn, G and M represent the daily (24-hour) values of net radiation, soil heat flux and energy storage in the canopy. The daily value of M is ignored in this model as it generally represents less than 5% of Sn in coniferous forests [13]. Thus, using the Sn values calculated for each layer according to the radiation interception submodel, PET is calculated separately for the canopy, understory and forest floor using equations (S3-S5), respectively.

$$PET_{\mathrm{Can}} = \alpha \frac{s}{s+\gamma} Sn_{\mathrm{Can}}\left(\frac{1}{L}\right) \qquad \text{(S3)}$$

$$PET_{US} = \alpha \frac{s}{s+\gamma} Sn_{US} \left(\frac{1}{L} \right) \quad \text{(S4)}$$

$$PET_{FF} = \alpha \frac{s}{s+\gamma} (Sn_{FF} - G) \left(\frac{1}{L} \right) \quad \text{(S5)}$$

In (Eq. S5) soil heat flux is estimated as a function of Sn_{FF} (Eq. S6) [7]. The default values for the a and b parameters are -43.1 and 0.335, respectively but may be adjusted the soil heat flux parameters if necessary.

$$G = a + b * Sn_{FF} \quad \text{(S6)}$$

Typically, the use of (Eq. S2) has been limited by the fact that, for dry canopy conditions, α must be calibrated against actual evapotranspiration rates for a particular site using Bowen ratio / energy balance techniques [19] which can be prohibitively expensive. For a range of dry forest conditions, α has been shown to vary from 0.6 to 1.1 [7]. However, for a wide range of relatively smooth, freely evaporating (wet) surfaces α is generally equal to 1.26 [16, 20, 5]. Values of α less than 1.26, typical in most forests with dry canopies, are thought to be the result of surface control of evaporation through stomatal resistance [11]. In an effort to eliminate the need for α to be experimentally determined for each new site, we suggest the use of a canopy resistance term (R_{Can}) based on mean literature values of α for dry canopies of various forest types (Table S2). Using this method α is set at 1.26 for wet canopy conditions and adjusted under dry canopy conditions (where evaporated water must pass through stomata) through the use of R_{Can}. The use of an approximated R_{Can} will undoubtedly lead to some error in the calculation of canopy PET, but it should provide a reasonable estimate suitable for the intended use of the model.

Table S2. Estimated canopy resistance (R_{Can}) for three forest types based on reported literature values of α'.

Forest Type	α'	$R_{Can} = 1 - (\alpha'/ 1.26)$	References
Dry Pine	0.7*	0.45	* Estimation based on reported range [19]
Douglas-fir	0.84	0.33	[3, 8, 19]
Broadleaf / understory	1.1*	0.13	[14, 19] * Estimation based on reported range

S1.4 Canopy water, throughfall and canopy evaporation

The amount of water held in the canopy at time t ($C_{Wat(t)}$) is calculated as the difference between incoming rainfall (P) and the sum of throughfall (TF) and canopy evaporation (E_{Can}) evaluated at each time step (dt) (Eq. S7). The fraction of rainfall intercepted by the canopy is determined as a function of canopy vegetation area index (VAI), where a maximum canopy storage term is calculated based on the assumption that at saturation the upper surface of leaves and branches can hold a film of water 0.2 mm in depth (Rutter, 1975), thus $C_{Max} = 0.2 * VAI$.

$$C_{Wat(t+dt)} = C_{Wat(t)} + (P_{(t)} - TF_{(t)} - E_{Can(t)}) * dt \qquad (S7)$$

Throughfall and canopy evaporation are calculated as follows:

$$TF = 0 \qquad C_{Wat} < C_{Max}$$
$$TF = C_{Wat} - C_{Max} \qquad C_{Wat} \geq C_{Max} \qquad (S8)$$

$$E_{Can} = \min(C_{Max}, C_{Wat}, PET_{Can}) \qquad (S9)$$

Stemflow is assumed to be included as part of throughfall.

S1.5 Soil water storage and drainage

Hydrologic dynamics in the forest floor and rooting zone are simulated using a multi-layered approach in which inflows and outflows are estimated sequentially for each soil layer (Figure S1). Water storage in and vertical movement through the mineral

soil layers are simulated using a "tipping bucket" type algorithm based on total porosity adjusted for coarse fragment content, field capacity and permanent wilting point boundaries (inputs to the model). Water stored in the humus and soil layers between field capacity and permanent wilting point boundaries is considered to be available for plant uptake. Water storage in the litter layer is calculated as a function of fine litter mass (i.e. not including coarse woody debris) per area and is assumed to be unavailable for plant uptake. Lateral flow or interflow of water from soil layers into stream channels is only allowed to occur when the water content of a given soil layer is greater than its estimated field capacity and is based on a coefficient related to slope.

S1.6 Soil-limited evapotranspiration

As the forest floor and soil layers begin to dry, evapotranspiration rates are limited by the availability of soil water. Transpiration loss is calculated separately from each layer for both the canopy and understory. Rooting depths of canopy and understory species are used to determine the layers from which water may be extracted. In order to represent the limited capacity of a drying soil to supply water to roots, particularly during periods of high PET, the daily, energy-limited transpiration rate is scaled back through the use of a relative transpiration rate (RTR) term. RTR is estimated as an empirical function of the percent of available water in each layer and PET [6] (Fig. A3). A root fraction term is also introduced for both the canopy (RF_{Can}) and understory (RF_{US}) as a scalar to account for the fraction of each layer occupied by roots.
This term is calculated from root depth occupancy data as well as soil layer depths provided by the user.

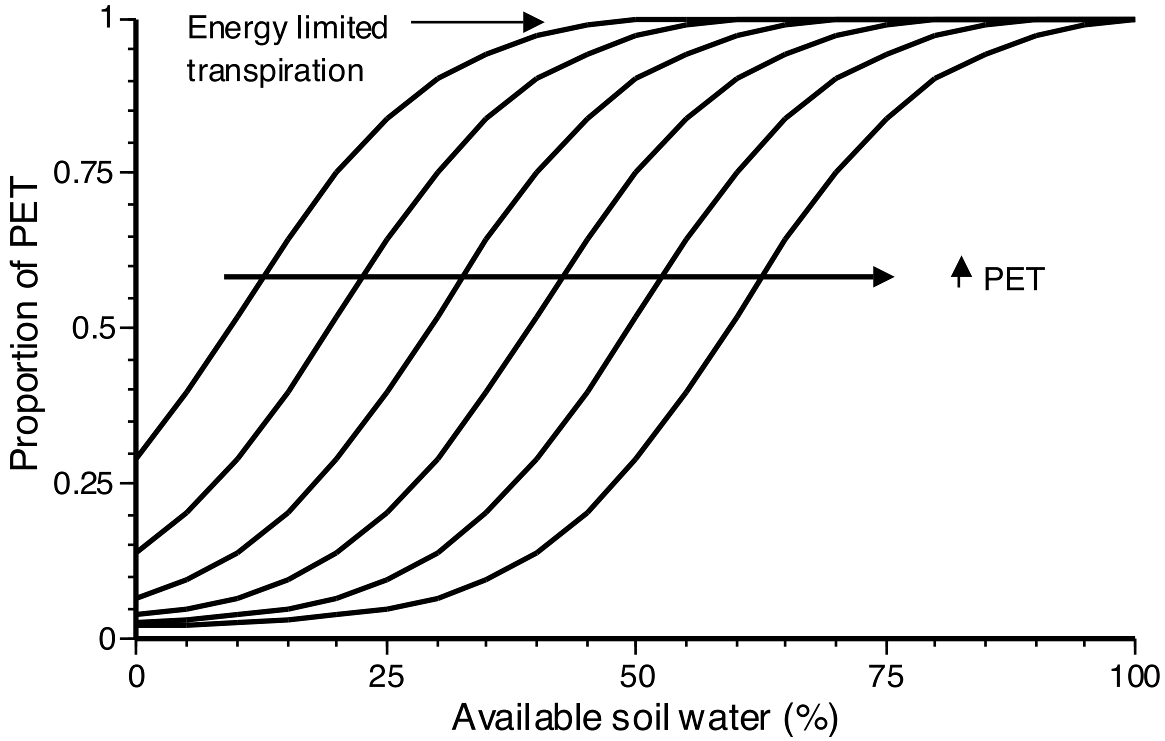

Figure S3. The relative rate of transpiration determined as a function of available soil water and energy-limited evapotranspiration (PET). The function curve shifts to the right as PET increases.

The daily extraction of water from the various layers via transpiration occurs sequentially from the top downward, beginning with the humus layer and continuing down through the soil B layer. The total amount of energy available for canopy transpiration ($CanT_{(total)}$) is estimated after accounting for the canopy PET energy consumed by canopy evaporation and canopy resistance (Eqs. S10-S11). $CanT_{(total)}$ is then used to drive transpiration loss from each of the soil layers using an energy budget approach, moving from the humus layer downward as the amount of extractable soil water is depleted in each soil layer (Eqs. S12-S14). The daily extractable fraction of available water in a given layer is largely a function of the estimated RTR value and is generally less than the total plant available water in that layer. The transpiration algorithm continues until either all layers have been depleted of extractable water or the energy available for daily transpiration is depleted. Understory transpiration is calculated simultaneously using the same method.

$$GCanT = (PET_{\mathrm{Can}} - E_{\mathrm{Can}}) \tag{S10}$$

where: GCanT = gross canopy transpiration (mm day $^{-1}$)

$$CanT_{(total)} = GCanT*(1-R_{Can}) \quad (S11)$$

$$CanT_{(humus)} = CanT_{(total)} * RTR_{(humus)}*RF_{Can(humus)} \quad (S12)$$

$$CanT_{(soilA)} = (CanT_{(total)} - CanT_{(humus)}) * RTR_{(SoilA)} * RF_{Can(soilA)} \quad (S13)$$

$$CanT_{(soilB)} = (CanT_{(total)} - (CanT_{(humus)} + CanT_{(soilA)})) * RTR_{(soilB)} * RF_{Can(soilB)} \quad (S14)$$

Energy-limited surface evaporation from the LF layer and humus compartments is also regulated as a function of water content through the use of a relative evaporation rate term (RER) estimated using a simple empirical function of available soil moisture. Drying proceeds from the top downwards (Eqs. S15 & S16) and evaporation from the humus layer is only allowed to occur when the litter layer has dried out.

$$E_{Litter} = PET_{FF} * RER_{(litter)} \quad (S15)$$

$$E_{Humus} = (PET_{FF} - E_{Litter}) * RER_{(humus)} \quad (S16)$$

S1.7 Canopy water stress

Typically, models of forest growth which include water stress as a feedback to forest growth have quantified tree water stress using a summed measure of soil water deficit. One of the problems in a using a summed water deficit for a measure of water stress is that it frequently fails to capture the dynamic interactions between different components of the hydrological cycle and net effect of such interactions on tree water stress. For example, as part of the Biology of Forest Growth Study near Canberra, Australia, [12] demonstrated that cumulative soil water deficit may not be well correlated with tree water stress as defined by pre-dawn xylem potential, a commonly used physiological indicator of tree water stress. The weakness of the technique is even greater when monthly summaries of climate data are used to calculate the water deficit.

In order to evaluate tree water stress more dynamically we propose the use of a cumulative index termed the transpiration deficit index (TDI) (Eq. S17-S18). TDI

provides a means of summarizing the net effect of several factors including canopy evaporation, understory transpiration and surface evaporation on actual tree transpiration. Furthermore the index is calculated on a daily timestep to better capture the cumulative effect of short term water stress events.

$$TDI = \sum_{i=0}^{i=t} \frac{CanT_{(\text{total})i} - CanT_{(\text{actual})i}}{CanT_{(\text{total})i}} \quad \text{(S17)}$$

$$CanT_{(\text{actual})} = CanT_{(\text{humus})} + CanT_{(\text{soilA})} + CanT_{(\text{soilB})} \quad \text{(S18)}$$

In (Eq. S17) the difference between total canopy transpiration demand ($CanT_{(total)}$) and actual canopy transpiration ($CanT_{(actual)}$) is divided by $CanT_{(total)}$ to normalize the effect of leaf area index on the relative canopy transpiration deficit. This adjustment effectively converts the total canopy water deficit to a per unit leaf area measure of water deficit which should be more representative of the water stress of individual trees.

S2. Supplemental References

1. Andrews SF, Flanagan LB, Sharp EJ, Cai T. Variation in water potential, hydraulic characteristics and water source use in montane Douglas-fir and lodgepole pine trees in southwestern Alberta and consequences for seasonal changes in photosynthetic capacity. Tree Physiol; 2012 32: 146-160.
2. Arp PA, Yin X. Predicting water fluxes through forests from monthly precipitation and mean monthly air temperature records. Can J For Res. 1992; 22: 864-877.
3. Black TA. Evaporation from Douglas-fir stands exposed to soil water deficits. Water Resour Res. 1979; 15: 164-170.
4. Coughlan JC, Running SW. Regional ecosystem simulation: A general model for simulating snow accumulation and melt in mountainous terrain. Landsc Ecol. 1979; 12: 119-136.
5. Davies JA, Allen CD. Equilibrium, potential, and actual evaporation from cropped surfaces in southern Ontario. Journal of Applied Meteorology. 1973; 12: 649-657.
6. Denmead OT, Shaw RH. Availability of soil water to plants as affected by soil moisture content and meteorological conditions. Agron J. 1962; 54: 385-390.
7. Flint AL, Childs SW. Use of the Priestly-Taylor evaporation equation for soil water limited conditions in a small forest clearcut. Agric For Meteorol. 1991; 56: 247-260.
8. Giles DG, Black TA, Spittlehouse DL. Determination of growing season soil water deficits on a forested slope using water balance analysis. Can J For Res. 1984; 15: 107-114.
9. Jarvis PG, James GB, Landsberg JJ. Coniferous forest. In: Monteith JL, editor. Vegetation and the Atmosphere, Vol 2, Case Studies. New York: Acedemic Press; 1976. pp 171-240.
10. Kelliher FM, Whitehead D, McAneney KJ, Judd MJ. Partitioning evaporation into tree and understory components in two young *Pinus Radiata* D Don stands. Agric For Meteorol. 1990; 50: 211-227.
11. McNaughton KG, Clothier BE, Kerr JP. Evaporation from land surfaces. In: Murray DL, and Ackro P, editors. Physical Hydrology: New Zealand Experience. Wellington North: New Zealand Hydrological Society; 1979. pp. 97-119.
12. Meyers BJ. Water stress integral—a link between short-term stress and long-term growth. Tree Physiol. 1988; 4: 315-323.
13. Monteith JL. Principles of Environmental Physics. London: Edward Arnold; 1973.
14. Munro DS. Daytime energy exchange and evaporation from a wooded swamp. Water Resour Res. 1979; 15: 1259-1265.
15. Nikolov NT, Zeller KF. A solar radiation algorithm for ecosystem dynamic models Ecol Modell. 1992; 61: 149–168.

16. Priestly CHB, Taylor RJ. On the assessment of surface heat flux and evaporation using large-scale parameters. Monthly Weather Review. 1972; 100: 81-92.

17. Rutter AJ. The hydrologic cycle in vegetation. In: Monteith JL, editor. Vegetation and the Atmosphere Vol. I Principles. London: Academic Press; 1975.

18. Spittlehouse DL, Black TA. A growing season water balance model applied to two Douglas-fir stands. Water Resour Res. 1981; 17: 1651-1656.

19. Spittlehouse DL, Black TA. Measuring and modelling forest evapotranspiration. Can J Chem Eng. 1981; 59: 173-180.

20. Stewart RB, Rouse WR. Substantiation of the Priestly and Taylor parameter a = 126 for potential evaporation in high latitudes. Journal of Applied Meteorology. 1977; 16: 649-650.

21. Warren JM, Meinzer FC, Brooks JR, Domec JC. Vertical stratification of soil water storage and release dynamics in Pacific Northwest coniferous forests. Agric For Meteorol. 2005; 130: 39–58.

22. Yin X, Arp PA. Predicting forest soil temperatures from monthly air temperature and precipitation records. Can J For Res. 1994; 23: 2521-2536.
